# Molecular and taxonomic characterization of arsenic (As) transforming *Bacillus* sp. strain IIIJ3–1 isolated from As-contaminated groundwater of Brahmaputra river basin, India

**DOI:** 10.1186/s12866-020-01893-6

**Published:** 2020-08-17

**Authors:** Soma Ghosh, Balaram Mohapatra, Tulasi Satyanarayana, Pinaki Sar

**Affiliations:** 1 Environmental Microbiology and Genomics Laboratory, Department of Biotechnology, Indian Institute of Technology Kharagpur, Kharagpur, 721302 India; 2 Present address: CSIR- National Environmental Engineering Research Institute, Kolkata Zonal Centre, Kolkata, 700107 India; 3 Present address: Department of Biosciences and Bioengineering, Indian Institute of Technology Bombay, Mumbai, 400076 India; 4 Department of Microbiology, University of Delhi South Campus (UDSC), New Delhi, 110021 India; 5 Presently affiliated to Department of Biological Sciences and Engineering, Netaji Subhas University of Technology, Sector 3 Dwarka, New Delhi, 110078 India

**Keywords:** Arsenic, *Bacillus cereus* group, Brahmaputra river basin, Ecophysiology, Dissimilatory reduction, Groundwater, Taxonomy

## Abstract

**Background:**

Microbe-mediated redox transformation of arsenic (As) leading to its mobilization has become a serious environmental concern in various subsurface ecosystems especially within the alluvial aquifers. However, detailed taxonomic and eco-physiological attributes of indigenous bacteria from As impacted aquifer of Brahmaputra river basin has remained under-studied.

**Results:**

A newly isolated As-resistant and -transforming facultative anaerobic bacterium IIIJ3–1 from As-contaminated groundwater of Jorhat, Assam was characterized. Near complete 16S rRNA gene sequence affiliated the strain IIIJ3–1 to the genus *Bacillus* and phylogenetically placed within members of *B. cereus* sensu *lato* group with *B. cereus* ATCC 14579(T) as its closest relative with a low DNA-DNA relatedness (49.9%). Presence of iC17:0, iC15:0 fatty acids and menaquinone 7 corroborated its affiliation with *B. cereus* group, but differential hydroxy-fatty acids, C18:2 and menaquinones 5 & 6 marked its distinctiveness. High As resistance [Maximum Tolerable Concentration = 10 mM As^3+^, 350 mM As^5+^], aerobic As^3+^ (5 mM) oxidation, and near complete dissimilatory reduction of As ^5+^ (1 mM) within 15 h of growth designated its physiological novelty. Besides O_2_, cells were found to reduce As^5+^, Fe^3+^, SO_4_^2−^, NO_3_^−^, and Se^6+^ as alternate terminal electron acceptors (TEAs), sustaining its anaerobic growth. Lactate was the preferred carbon source for anaerobic growth of the bacterium with As^5+^ as TEA. Genes encoding As^5+^ respiratory reductase (*arr* A), As^3+^ oxidase (*aio*B), and As^3+^ efflux systems (*ars* B, *acr3*) were detected. All these As homeostasis genes showed their close phylogenetic lineages to *Bacillus* spp. Reduction in cell size following As exposure exhibited the strain’s morphological response to toxic As, while the formation of As-rich electron opaque dots as evident from SEM-EDX possibly indicated a sequestration based As resistance strategy of strain IIIJ3–1.

**Conclusion:**

This is the first report on molecular, taxonomic, and ecophysiological characterization of a highly As resistant, As^3+^ oxidizing, and dissimilatory As^5+^ reducing *Bacillus* sp. IIIJ3–1 from As contaminated sites of Brahmaputra river basin. The strain’s ability to resist and transform As along with its capability to sequester As within the cells demonstrate its potential in designing bioremediation strategies for As contaminated groundwater and other ecosystems.

## Background

Arsenic (As) contamination in groundwater of Bengal Delta Plain (BDP) (covering large parts of Bangladesh and India) has become an emergent health concern for millions of people over the decades [1, 2]. Consumption of As contaminated drinking water and food grains has been implicated with severe health crisis including arsenicosis and cancer affecting more than 100 million people in BDP [3, 4]. The predominant inorganic species of As in naturally (geogenic) contaminated alluvial aquifers are As^3+^ and As^5+^. As^3+^ is more mobile in aqueous, oxic environments while As^5+^ tends to remain adsorbed to the sediments in anoxic state [5]. Relative abundance of these two species which eventually affects As -mobility and –toxicity in aquifers depends mainly on the prevailing redox conditions and inhabitant microbial activity [6–9]. The resident microorganisms in contaminated aquifer involved in redox transformation of As are considered to be the most precarious factors for As-release into the groundwater [1, 10–13]. Eco-physiological, taxonomic, and molecular characterization of As transforming bacteria from As contaminated groundwater constitute an important component of subsurface geomicrobiology, particularly to better understand the potential of these organisms in geo-cycling of As and their natural attenuation [13–16].

In recent years, several geomicrobiological studies have unanimously agreed upon the wide physiological role of taxonomically diverse bacterial populations viz. *Alpha*-, *Beta*-, *Gamma*-*proteobacteria*, *Firmicutes* (*Bacillus* and relatives), *Actinobacteria*, etc. influencing As bio-geochemistry in alluvial groundwater [13, 17–23]. Arsenic transforming bacteria have been known to deploy an array of metabolic routes including lithotrophic to heterotrophic mechanisms of As-oxidation [24], -reduction [25], -respiration [26], and -methylation [27], affecting As -solubility, -speciation, and -mobilization. Heterotrophic As^3+^ oxidizing (HAO) and chemoautotrophic As^3+^ oxidizing (CAO) members have been described to use As^3+^ as their electron source [28] and dissimilatory As^5+^ respiring members (DARB) to use As^5+^ as electron acceptor [29]. Among these organisms, DARB have been identified to play the crucial role in As mobilization from As bearing host minerals in alluvial aquifers [8, 16, 30–32]. With respect to the taxonomic and physiological characterization of DARB from diverse habitats, till date 32 cultivable representatives have been studied [29, 32, 33]. Interestingly, only few members of *Proteobacteria* i.e. *Desulfuromonas*/ *Pelobacter* sp. WB-3 [32], *Rhizobium arsenicireducens* KAs 5-22^T^ [16], *Pseudoxanthomonas arseniciresistens* KAs 5-3^T^ [34]; *Achromobacter* sp. KAs 3-5 [35] and *Firmicutes viz. B. arsenicus* [36], *B. indicus* [37] have been isolated from BDP (West Bengal) and characterized thoroughly. Recently, an As tolerant siderophore producing *Staphylococcus* sp. strain TA6 has been isolated from upper Brahmaputra River Basin (BRB) aquifer and reported to have possible role in biogeochemical cycling of As therein [38]. However, except few recent documentations, the geomicrobiology of the vast As rich alluvial aquifers of BRB remain largely unexplored [38–40].

Using anaerobic microcosms of subsurface As bearing sediment of BRB we have recently demonstrated the prominent role of *Bacillus* and other members of *Firmicutes* in As/ Fe reduction and As mobilization [40]. Particularly, strain IIIJ3-1 has been recently shown to play prominent role in As mobilization from sediments of BRB aquifer under NO_3_^-^ amended anaerobic microcosm by the process of oxalate mediated mineral weathering [41]. However, detailed molecular and taxonomic characterization of any of the As reducing taxa remained still elusive. In general, presence of *Bacillus* spp. in As-contaminated groundwater of Bengal basin and other alluvial aquifers as well in various polluted environment and their As transformation abilities have been well documented [17, 42–45]. Dissimilatory reduction of As^5+^ by *Bacillus* spp. from soda lake, mono lake, mine environment, and effluent plants (*B. arsenicoselenatis*, *B. selenitireducens*, *B. macyae* and *B. selenatarsenatis*) and As^3+^ oxidation (*B. firmus* L-148, *Bacillus* sp. PNKP-S2) have been described [36, 37, 46–49]. Metabolic versatility including the ability of dissimilatory reduction of Fe and As or even SO_4_ by the members of this taxon could have strong influence of subsurface As mobility. *Bacillus* strains isolated from As contaminated sites of West Bengal (India) [36, 37], or Datong basin (China) [43] and Hetao basins (Mongolia) [50] were characterized. However, till date, no pure culture *Bacillus* strain capable of dissimilatory As reduction has been isolated from the vast As contaminated area of North East India (covering the BRB) and studied for its taxonomic characterization, overall physiology and As biotransformation ability (including respiratory function). The present study was carried out to highlight the taxonomic description of an As^5+^- respiring strain IIIJ3-1isolated from As-contaminated groundwater of BRB, India.

In the present study, we have reported a novel bacterium strain IIIJ3-1, belonging to the *B. cereus* group isolated from As contaminated groundwater of Jorhat, Assam (India) capable of As^3+^ oxidation as well as dissimilatory As^5+^ reduction. Molecular phylogenetic analysis coupled with chemotaxonomic and ecophysiological characterization and study on As biotransformation properties have been performed to establish its taxonomic novelty within the group *B. cereus sensu lato*. Based on its ability to survive under both oxic and anoxic environments and high As resistance as well as As redox transformation, the strain has been chosen for further investigation and characterization of its eco-physiological property.

## Results

### Polyphasic taxonomic characterization of strain IIIJ3–1

#### 16S rRNA gene phylogenetic analysis

**Fig. 1:**
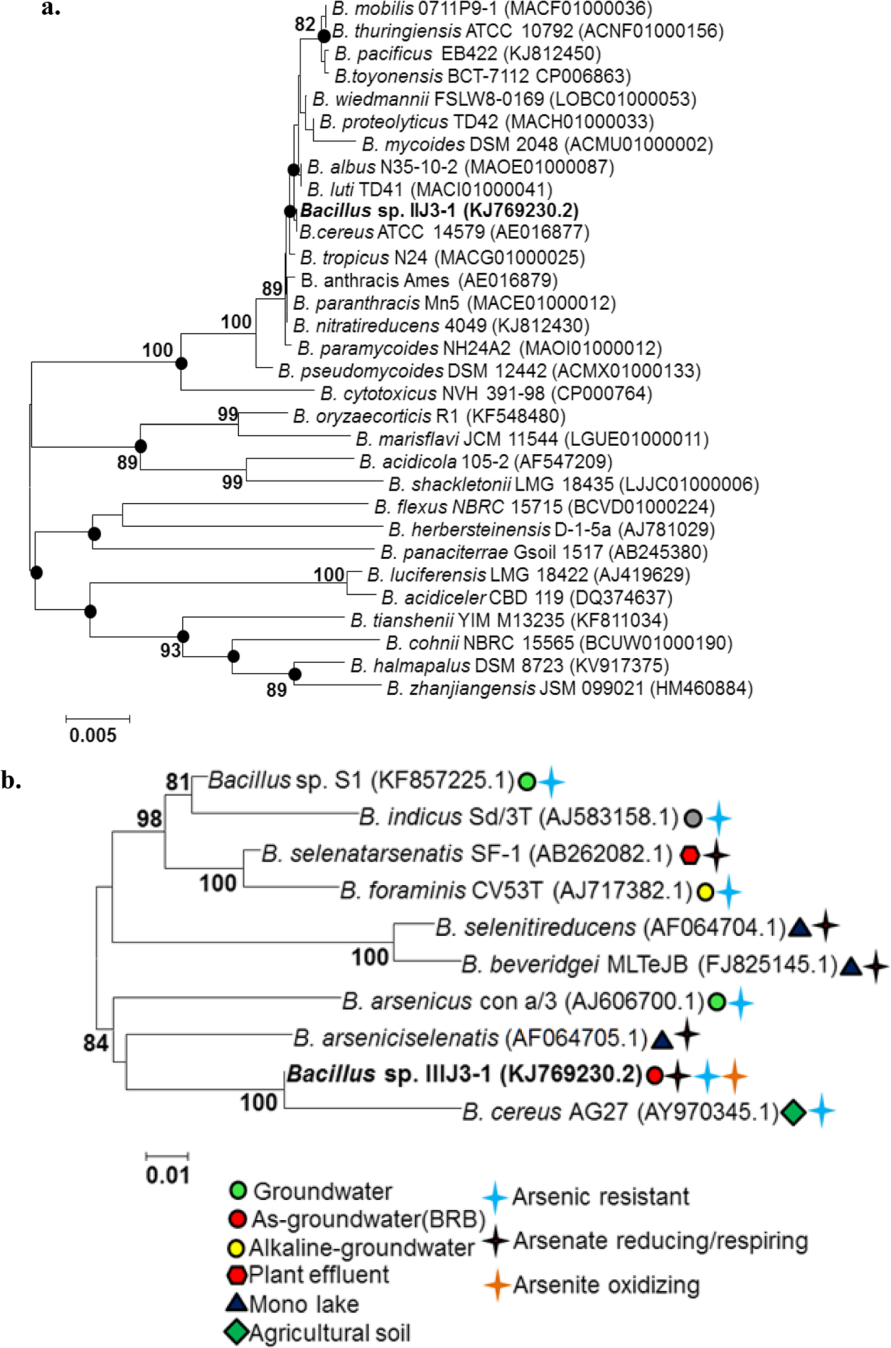
Unrooted Neighbour Joining (NJ) phylogenetic tree based on 16SrRNA gene sequences of strain IIIJ3–1 and related *Bacillus* spp., **a.** consensus phylogenetic tree of strain IIIJ3–1 and all validly described type strains of *Bacillus* using the Jukes-Cantor distance model considering a total of 1485 positions in the final dataset, **b.** Phylogenetic relationship of strain IIIJ3–1 and other As transforming *Bacillus* spp. isolated from diverse As-impacted habitat with a total of 1490 positions in the final dataset. The black solid circle indicates the consistent tree topology obtained through both NJ and ML methods. Bootstrap values (percentage of 1000 replications) greater than 60% are represented at the nodes. The GenBank accession numbers are mentioned in the parentheses. Scale bar denotes the rate of nucleotide substitution

Comparison of near complete 16S rRNA gene sequence (1474 bp) of strain IIIJ3-1 indicated high degrees of sequence similarities (98.2-99.9 %) to the members of the genus *Bacillus*, where type strain *B. cereus* ATCC 14579(T) was the most hit taxon (99.9 % similarity) at 99 % query coverage. The NJ phylogenetic reconstruction showed that strain IIIJ3-1 formed a coherent cluster of monophyletic pattern with the type strain of *B. cereus* ATCC 14579(T) and claded to the type members of *Bacillus* (Fig 1a), indicating its affiliation to the genus *Bacillus*. Both ML and ME phylogenetic reconstruction methods indicated a consistent tree topology clading strain IIIJ3-1 to the *B. cereus* ATCC 14579(T) as the nearest phylogenetic neighbor. Phylogenetic analysis involving As transforming *Bacillus* spp., from various habitat showed that strain IIIJ3-1 is taxonomically closest to the *B. cereus* AG27 (AY970345.1, an As-resistant bacterium from agricultural soil) [51], denoting its species level affiliation to the *B. cereus* members (Fig 1b). While, other *Bacillus* members having arsenate and selenite respiratory/ reductive activities (*B. arseniciselenatis* from mono lake and *B. arsenicus* from groundwater) coherently clustered with the clade comprising strain IIIJ3-1.

#### Genomic analysis

Genomic G+C content of strain IIIJ3-1 was found to be 45.0 mol%. The value of ∆T_m_ for the heteroduplex, formed with the genomic DNA of test isolate IIIJ3-1 and its closest phylogenetic match *B. cereus* ATCC 14579(T) at optimal renaturation temperature (T_OR_) 69.95°C, and homoduplex of *B. cereus* ATCC 14579(T) was found to be 8°C (Supplementary Fig S1). Relative binding ratio indicating DNA-DNA relatedness of the two strains was found to be 49.9%.

#### Chemotaxonomic characteristics

Cellular FAME profile of strain IIIJ3-1 showed abundance of isoC_17:0_ (10.3%), isoC_15:0_ (8.8%) followed by C_15:0_ (6.8%), C_17:0_Δ (5.7%), and 2OH-C_14:0_ (5.2%) (Supplementary Fig. S2). Relatively less abundant 2-OH-C_16:0_(3.8%), C_18:2_^9,12^ (3.2%), C_19:0_ (3.2%), C_11:0_ (3.1%), trans C_18:1_^9^(2.9%), cis-C_18:1_^9^ (1.8%), C_16:0_ (2.7%), isoC_16:0_ (2.4%), and anteiso-C_15:0_(2.4%) were detected. Trace amounts of 2OH-C_10:0_, 3OH-C_12:0_, 3OH-C_14:0_, C_14:0_ and unresolved peaks corresponding to 24% of total FAMEs were obtained. Detailed analysis of isoprenoid respiratory quinones revealed presence of several menaquinones (MKs) in strain IIIJ3-1 (Table 1). Abundance of MK-5 (55%), MK-6 (25%), and MK-7 (7.8%) fractionated by HPLC at 2.9, 4.4 and 8.4 minutes were determined (Supplementary Fig. S3a). MALDI-MS analysis followed by Pubchem database search revealed the molecular identity of the MKs. (Supplementary Fig. S3b). Derivatives of MK-5 were found to be MK-5-d7, MK-5 epoxide and demethyl MK-5 d7 with corresponding molecular masses of 519, 535, 505 respectively. MK-6 derivatives were found to be methyl substituted MK-6 d5, MK-6 d5 and deoxygenated MK-6 with molar mass of 599, 585 and 551.56, respectively. MK-7 d7 and MK7-d7 epoxide with molar mass of 656 and 672 were found to be the major MK-7 derivatives (Supplementary Fig. S3c).

**Table 1:** Differential phenotypic and biochemical characteristics of *Bacillus cereus* strain IIIJ3–1 and taxonomically and metabolically related species.

	1	2	3	4	5	6	7	8	9	10	11	12	13	14
Colony colour/ nature	cream	ND	Red	Cream	Brown	Yellow–orange	cream	rhizoidal	white	White/grey	cream	rhizoidal	White	cream
As^5+^ tolerance (mM)	350	0	NA	20	0	20	nr	nr	nr	nr	nr	nr	nr	nr
As^3+^ tolerance (mM)	10	0.3	0.3	0.3	0	0.3	nr	nr	nr	nr	nr	nr	nr	nr
Catalase	+	+	+	+	nr	nr	+	+	+	+	nr	+	+	+
Oxidase	+	+	–	+	–	–	nr	–	+	–	nr	–	–	+
Temperature Range (^°^C)	20–50	nr	nr	20–40	18–37	15–37	20–50	10–40	10–45	10–45	5–37	5–37	10–50	10–45
Salinity (%)	0–4	2.4–6	0–6	0–1	0–2	0–2	nr	0–2.5	0	0	nr	0–4	nr	0–5
pH range	6–10	8.5–10	8.5–10	5.5–8	7–9.5	06–7.1	nr	5–9.5	5–9.5	5–9.5	nr	5–9.5	nr	5–9.5
(G + C)%	45	40	49	35	nr	41.2	35.8	34–36	35.3	35.38	35.5	34.1	35.4	35.6
Major quinone	MK5, MK6, MK7	nr	nr	MK7	MK7	MK7	nr	nr	MK7	MK8 MK7 MK3	nr	MK7	MK7	nr
Mannitol	–	nr	nr	+	–	–	–	–	–	–	–	–	–	–
Rhyzoidal colony	–	–	–	–	–	–	–	+	–	–	–	+	–	–
Starch hydrolysis	+	+	+	+	+	+	–	–	+	+	+	+	+	+
Glycerol	–				–	nr	–	+	–	–	+	–	–	–
Ribose	ND	nr	nr	–	+	+	+	+	+	–	+	+	+	+
Galactose	–	–	+	nr	–	nr	–	–	–	–	–	–	–	–
D-mannose	–	nr	nr	nr	+	+	+	–	–	+	–	–	–	–
N acetyl Glucosamine	+	nr	nr	nr	+	nr	+	+	+	+	+	+	+	+
Salicin	–	nr	nr	nr	–	nr	+	+	+	+	+	+	–	+
Cellobiose	–	nr	nr	–	–	+	+	–	+	+	+	+	–	+
Sucrose	+	nr	nr	+	–	+	–	–	+	+	–	+	+	+
Trehalose	+	nr	nr	nr	+	nr	–	+	+	+	+	+	+	+
Glycogen	ND	nr	nr	nr	+	nr	–	+	+	+	+	+	+	+
β-gentiobiose	–	nr	nr	nr	+	nr	–	–	–	–	–	–	–	–
Turanose	–	nr	nr	nr		nr	–	–	–	–	–	–	+	–

Negative growth results were found for sugars- D-raffinose, mellibiose, D- mannose, D- galactose, β-gentiobiose, D-turanose, α-rhamnose, 3-methyl glucose, D-fucose and β–methyl–D–glucoside, sugar alcohols- D-sorbitol, D- mannitol, D-arabitol, myo-inositol, glycerol, sugar amine and amide- N-acetyl galactosamine and glucuronamide, amino acids- D-serine, α-glutamic acid, α-histidine glycyl-L- proline, L-alanine, L-arginine, sugar alcohols- glycerol, D- salicin, sugar acids- p-hydroxy phenyl acetic acid, L-aspartic acid, D-galactouronic acid, L-galactonic acid, D-glucuronic acid, D-lactic acid methyl ester, citric acid, γ-amino butyric acid, α-hydroxy butyric acid, β-hydroxy D-Lbutyric acid, α-keto butyric acid, N-acetyl neuraminic acid, D-aspartic acid, α- pyroglutamic acid, quinic acid, acetic acid, fusidic acid, D- saccharic acid, bromosuccinic acid, formic acid, nalidixic acid, sodium butyrate, mucic acid, α-ketoglutaric acid, D-malic acid, acetoacetic acid, propionic acid and Tween 4

*B. inferioriaquae* strain IIIJ3–1 (present study)*;* 2. *B. arsenicoselenatis;* 3. *B. selenatireducens* (2,3) [46]*;* 4. *B. arsenicus* [36]*;* 5. *B.barbaricus* [52]; 6*. B. indicus* [37]; 7. *B. cytotoxicus;* 8. *B. pseudomycoides;* 9. *B. cereus;* 10. *B. thuringiensis;* 11. *B. weihenstephanensis;* 12. *B. mycoides;* 13. *B.anthracis* (7–13) [53]*;* 14. *B. toyonensis* [54]. Symbols; (+) = positive, (−) = negative; ND = not determined, nr = no report, MK = menaquinone

#### Phenotypic and biochemical characterization

**Fig. 2:**
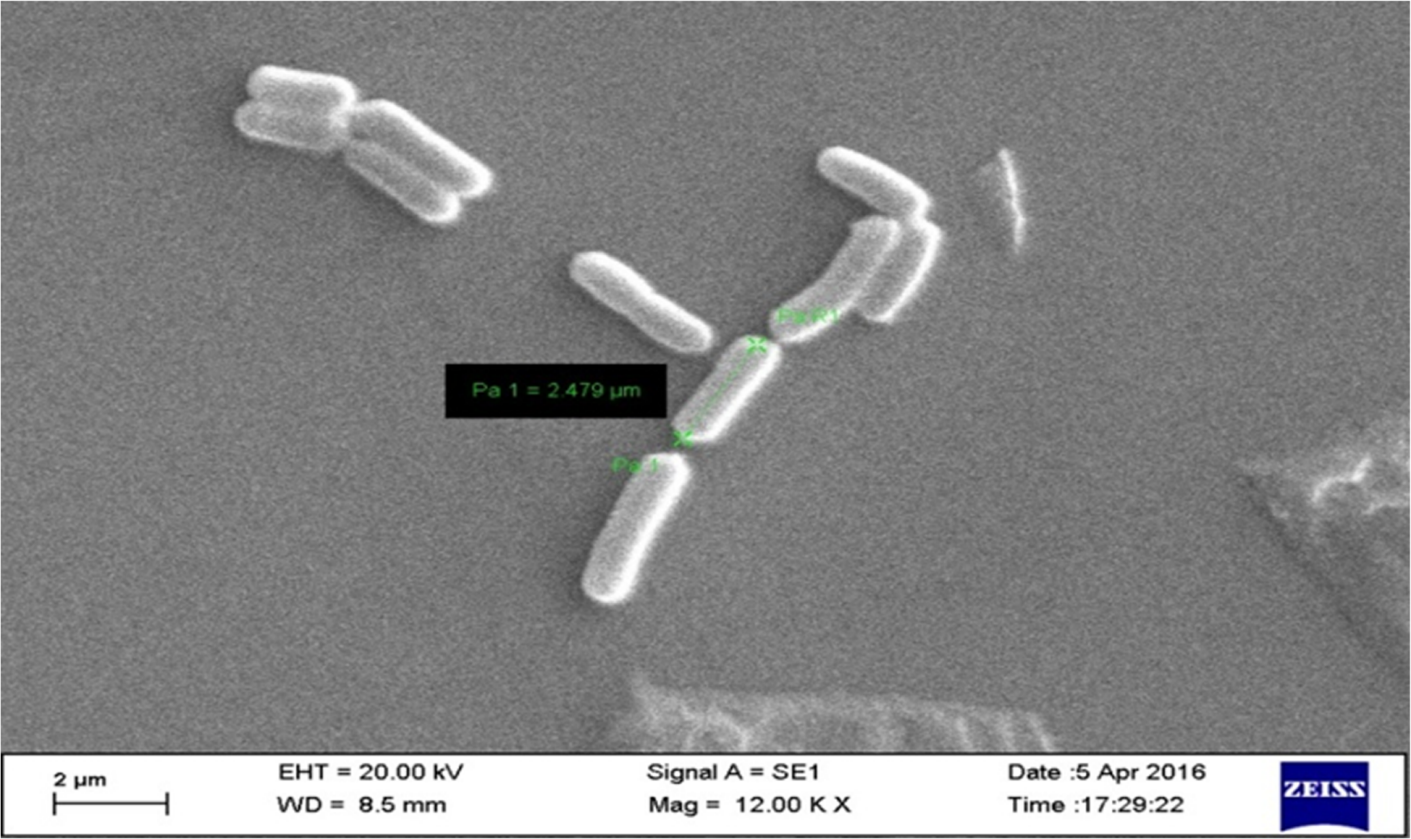
Scanning electron micrograph of strain IIIJ3–1(T) after 18 h of growth on LB agar plate

The colonies of strain IIIJ3-1 were creamy white with undulating edges and pasty appearance. The colonies became rhizoidal after 48h of growth. Scanning electron micrograph confirmed the cells of strain IIIJ3-1 to be rod-shaped with cell size of 2.5 x 0.7 μm (Fig. 2). The strain was found to be Gram-positive, facultative anaerobic, non-motile, catalase-, and oxidase- positive, endospore-forming (terminal to sub-terminal), non-flagellated, capsulated, and capable of reducing As^5+^ via dissimilatory process. Differential physiological characteristics of strain IIIJ3-1 with those of phylogenetically related *Bacillus* spp. including those isolated from As contaminated groundwater have been summarized in Table 1. Ability to utilize diverse C-sources and TEA of strain IIIJ3-1 was ascertained and compared with other *Bacillus* spp. reported to be As resistant (Supplementary Table 1). Anaerobic growth of strain IIIJ3-1 with diverse TEAs yielded positive response with As^5+^, SO_4_^2-^ , Fe^3+^, NO_3_^-^ and Se^6+^.

### Arsenic transformation potential of strain IIIJ3–1

#### Aerobic As biotransformation assay

The activity of resting cells of strain IIIJ3-1 for substrates As^5+^ and As^3+^ was investigated. Using molybdenum blue method, the K_m_ for As^5+^ reduction was estimated to be 10 mM (Supplementary Fig. S4) with a V_max_ of 0.25 mM h^-1^ and those for As^3+^ oxidation were found to be 2.8mM and 0.2 mM h^-1^, respectively. Kinetics for As^5+^ reduction and As^3+^ oxidation by strain IIIJ3-1 demonstrated its considerably higher affinity towards As^3+^ (~3 times) than As^5+^ (Supplementary Fig. S4).

#### Anaerobic growth of strain IIIJ3–1

**Fig. 3:**
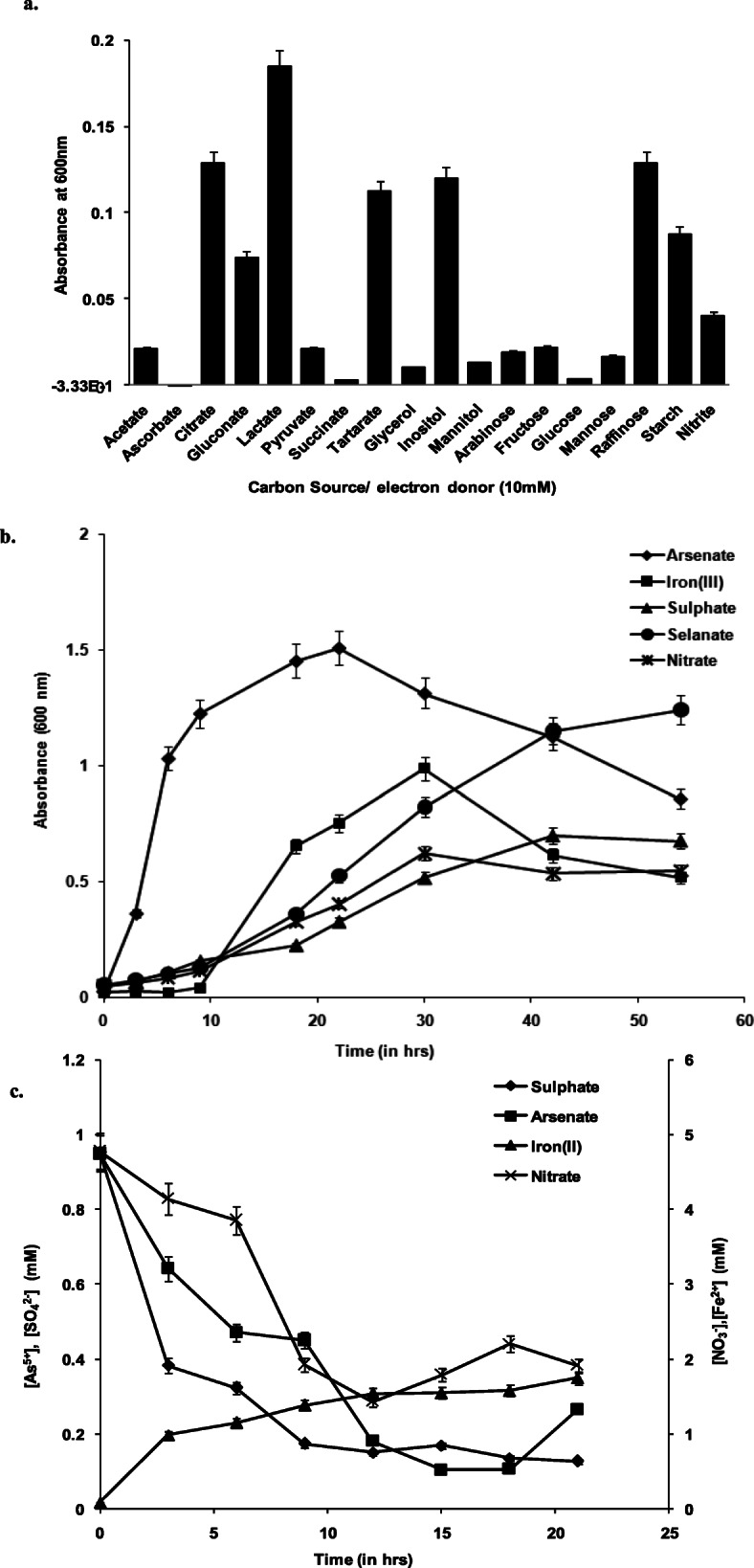
Anaerobic growth kinetics of strain IIIJ3–1 in presence of alternate carbon source and terminal electron acceptors. **a.** C- source/e^−^ donor utilization profile with As^5+^ as TEA, **b.** anaerobic growth kinetics in presence of alternate e^−^ acceptors, and **c.** reduction profile of alternate electron acceptors. Error bars indicate 5% percent of the value plotted

Anaerobic growth of strain IIIJ3-1 with varying TEAs revealed that As^5+^ was the most respiratory substrate preferred (with lactate as electron and C- source) followed by utilization of other TEAs in the following order: Fe^3+^ > Se^6+^> SO_4_^2-^> NO_3_^-^ (Fig. 3a). No growth could be detected with S_2_O_3_^2-^. Mean generation time (*g*) and mean growth rate constant (k) of the strain with As^5+^ was 45 minutes and 0.220, respectively followed by increasing *g* and decreasing *k* for Fe^3+^, Se^6+^, NO_3_^-^, and SO_4_^2-^ (Supplementary Table 2) indicating fastest growth utilizing As^5+^ as compared to others. Electron donor utilization profile with As^5+^ as TEA revealed maximum cell yield with lactate followed by raffinose, citrate, inositol, tartarate, gluconate, starch, nitrite and pyruvate. Faint or negligible growth was found with mannose, fructose, acetate, arabinose, succinate, mannitol, glucose and glycerol (Fig. 3b). Reduction profiles for As^5+^, Fe^3+^, NO_3_^-^ and SO_4_^2-^ by strain IIIJ3-1 indicated near complete transformation of As^5+^ within 15 h of growth (Fig. 3c). Linear regression and correlation analysis (Supplementary Fig. S5) of growth vs coupled reduction profile (Conc^-1^) showed that a high goodness of fit (R^2^= 0.8, r = -0.671) was achieved for SO_4_^2-^ reduction w.r.t time indicating the stoichiometric balance between reduction profile and energy generation by IIIJ3-1 for cellular growth. While, a high variance (R^2^=0.38, r = -0.915, R^2^=0.38, r = 0.691) was noted for both As^5+^ and Fe^3+^ indicated a partially uncoupled behavior of growth vs reduction profile (Conc^-1^). This observed stoichiometric imbalance between reduction profile and energy generation by IIIJ3-1 might be attributed to the cellular As uptake during reduction/respiration by IIIJ3-1. A relatively less variance (R^2^= 0.46, r = -0.705) was noticed for NO_3_^-^ reduction w.r.t time indicated a partially coupled growth behavior of cells of IIIJ3-1, which might be attributed to the organization of denitrification pathway i.e. either only reduction of NO_3_^-^ to NO_2_^-^ or denitrification to NH_4_^+^ or N_2._ The overall observation indicated the versatile ability of the bacterium to cope up with wide redox fluctuation within its natural environment *i.e.,* groundwater.

#### Molecular analysis

**Fig. 4:**
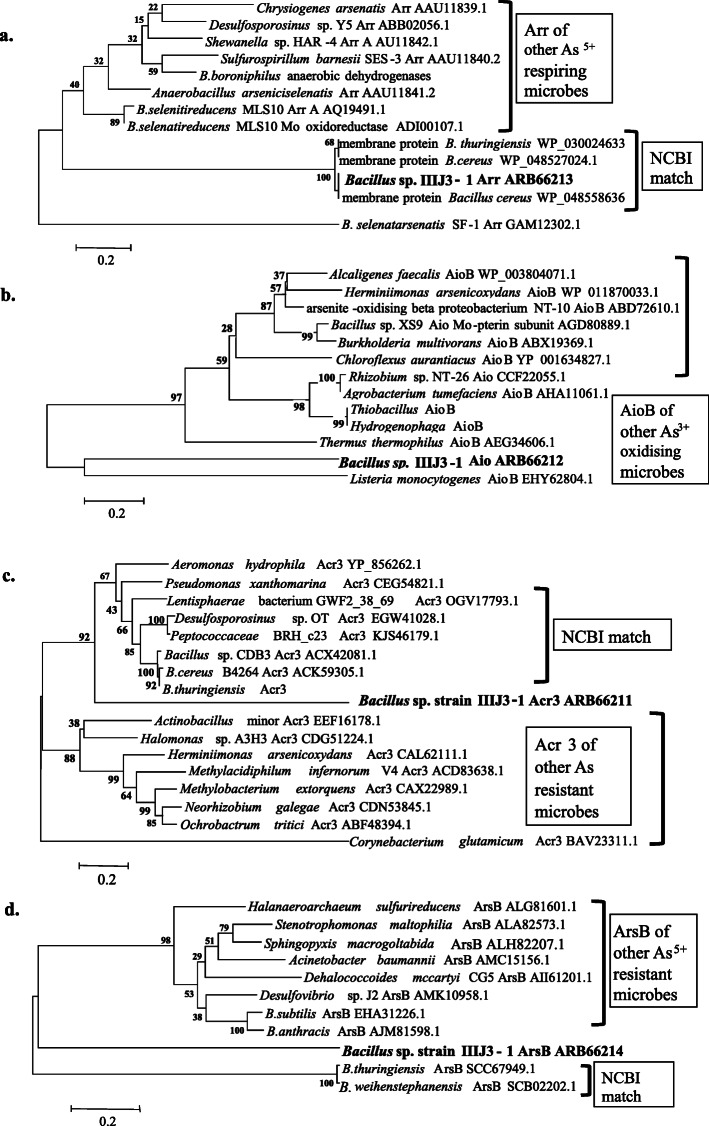
Maximum Likelihood phylogenetic tree for As homeostasis genes based on their deduced amino acid sequences: Arr (**a**), AioB (**b**), Acr3 (**c**) and ArsB (**d**)

Presence of As homeostasis genes *arr*A*, aioB, ars*B and *acr3* (1) were observed through PCR amplification with respective primers, sequencing and phylogenetic analysis. The *arr*A *gene* (147 deduced amino acids) from strain IIIJ3-1 revealed 99% identity with membrane proteins of *B. cereus.* Close phylogenetic relatedness of *arr* from strain IIIJ3-1 with the hypothetical membrane proteins of members of *B. cereus* group (Fig. 4a). Multiple alignment of the deduced amino acid sequences of putative Arr from strain IIIJ3-1 and Arr from other DARBs indicated 12 conserved amino acid residues and 6 residues replaced by amino acids bearing similar side chain or same functional group maintaining the expected role (Supplementary Fig. S6a).

NCBI database search for the deduced amino acid sequences of putative AioB from strain IIIJ3-1 did not reveal sequence identity with any of the known Aio sequences. However, phylogenetic analysis of AioB sequence obtained from strain IIIJ3-1 indicated its distant relatedness with As^3+^oxidase reported from *Listeria monocytogenes* (Fig. 4b). Multiple alignments of 188 deduced amino acids of AioB from strain IIIJ3-1 with 12 other reported AioB sequences revealed 19 consensus amino acids with a conserved arginine residue (Supplementary Fig. S6b). The conserved regions FDHGG, GGGFEN and IHNRPAYNSE of known As^3+^ oxidases were found to be 40%, 17% and 20% conserved in the putative AioB sequence of IIIJ3-1 respectively.

Deduced amino acid sequences of *acr*3 and *ars*B genes showed a similarity match with arsenical resistance proteins and arsenite pump proteins (ArsB) of various As resistant strains, respectively. Phylogenetic analysis of the deduced amino acid sequences of Acr3 obtained from *B.cereus*IIIJ3-1 showed relatedness with the Acr3 sequence obtained from other members of *B. cereus*, *Desulfosporosinus, Peptococcaceae, Lentisphaera, Pseudomonas* and *Aeromonas* (Fig. 4c). However, a bootstrap value of only 67 showed significant sequence diversification of the putative Acr3 obtained from IIIJ3-1. Deduced amino acid residues of the putative ArsB from strain IIIJ3-1 revealed comparatively closer phylogenetic relatedness to those reported from *Halanaeroarchaeum,* followed by ArsB sequences of *Stenotrophomonas, Acinetobacter, Desulfovovibrio, B. subtilis* and *B. anthracis* than those obtained from members of *B. cereus* (*i.e. B. thuringiensis*, *B. weihenstephanensis* (Fig. 4d). Multiple alignments of the deduced amino acid sequences of *acr3* (94a.a) and *arsB* (107a.a) showed that the sequences were 35% and 21% conserved, respectively (Supplementary Fig. S6c and d).

### SEM and EDX analysis

**Fig. 5:**
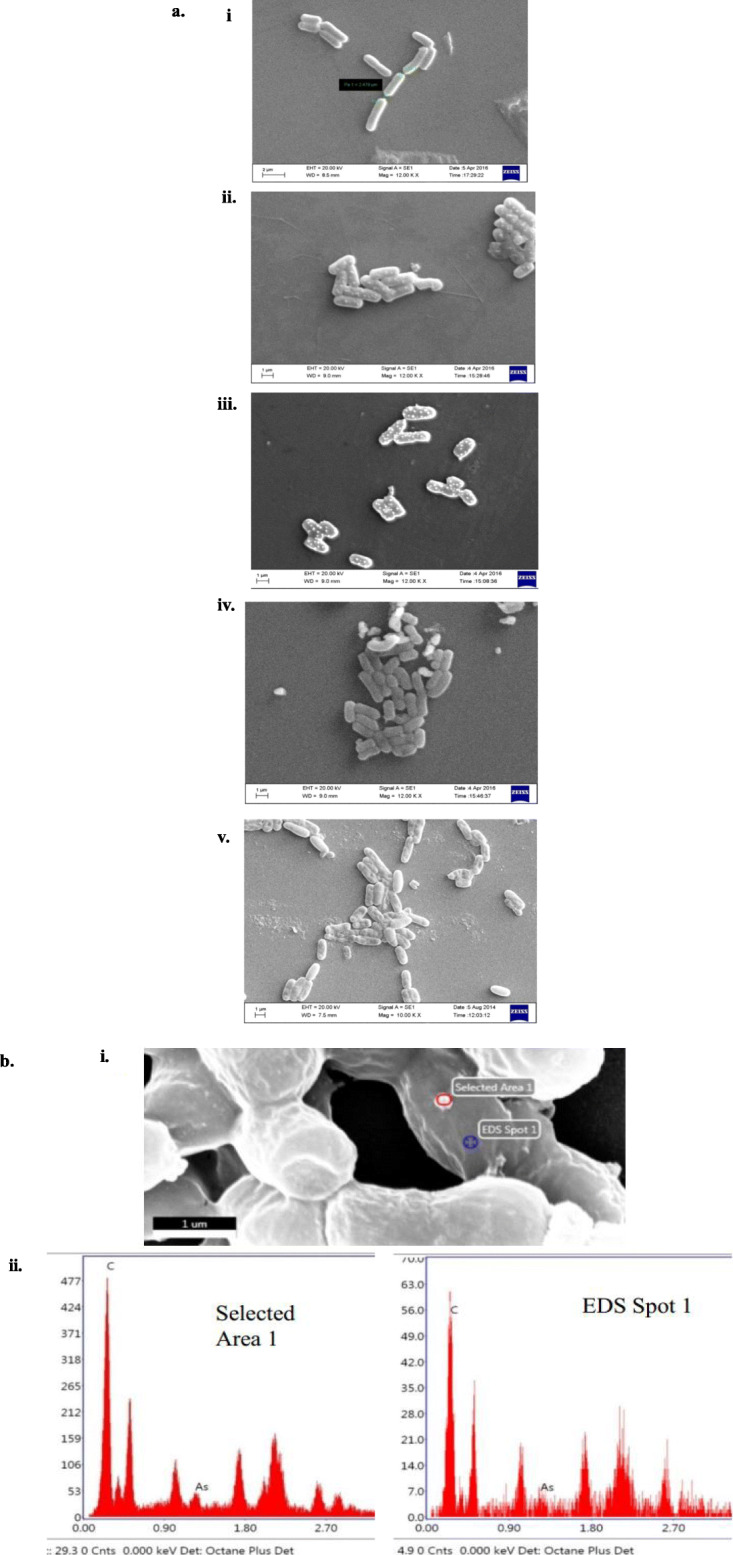
Cells grown **(i)** without As stress i.e. control **(ii)** aerobically with As^3+^,**(iii)** anaerobically with As^3+^, **(iv)** aerobically with As^5+^, **(v)** anaerobically with As^5+^**(a)**. Analysis of electron dense particles **(i)** magnified image of anaerobically grown strain in presence of As^3+^ **(ii)** point EDX analysis on an electron opaque dot and on another point (as control) on cell surface of strain IIIJ3–1 (**b**)

Scanning electron microscopic images revealed distinct morphological changes in terms of cell shape and size of strain IIIJ3-1 following growth with As^3+^ or As^5+^ under aerobic and anaerobic conditions (Fig. 5a). Control cells were found to be ~ 3 μm x 1 μm with smooth cell surface (Fig. 5a i) whereas those grown with As showed reduction in cell size and exhibited rough and convoluted cell surface. Cells grown with As^3+^ (under both aerobic and anaerobic conditions) indicated electron opaque dots and reduction in length but increase in diameter (i.e 2.4 μm X 1.4 μm for aerobically grown cells and 2.2 μm X 1.2 μm for anaerobically grown cells). Cells under As^5+^ stress (both aerobic and anaerobic) did not show distinct As rich dots but cell shrinkage was prominent i.e 2 μm X 0.4 μm and 1.8 μm X 0.5 μm aerobically and anaerobically grown cells respectively. Moreover, the As stressed cells were found to be clustered together and covered with an exopolysaccharide layer which contributes to effectively lower the exposed cell surfaces through which the cells adhere to each other (Fig. 5; Table 2). Surface area: volume ratio for control cells is found to be 4.67 which eventually decrease for As^3+^ stressed cells to 3.7 and 4.2 for oxic and anoxic growth, respectively. However, the As^5+^ cells show an increase in surface area : volume ratio upto 6 and 5 for oxic and anoxic growth, respectively along with prominent surface constrictions and clustering of the cells. The pattern of As accumulation as determined from the EDX data is as follows (Fig. S7): As^3+^ grown aerobic cell (1.21 wt%) > As^3+^ grown anaerobic cells (0.94 wt%) > As^5+^ grown aerobic cells (0.36 wt %) > As^5+^ grown anaerobic cells (0.09 wt %) (Supplementary Fig. S7 a-d). This observation corroborated the stoichiometric imbalance between growth and reduction kinetics profile by the bacterium. No peak for As could be observed for control cells (Supplementary Fig. S7e). Point EDS analysis performed on electron opaque dots formed during incubation of the cells with As^3+^ showed prominent peak for As. Such a peak for As was found to be absent on any other position on the cell surface except on the dots formed (Fig. 5b). Carbon content of the As containing electron opaque dots was found to be much higher than that of the normal cell surface which might have occurred due to encapsulation of the dots with polymeric C containing exudates.

**Table 2:** Morphometric calculations of strain IIIJ3–1 grown with and without As stress; h = length, r = radius, V = volume and, A = area of the cells

Growth condition	h (μm)	r (μm)	V (μm^3^)	A (μm^2^)	A/V
Control	3.0	0.5	2.4	11.0	4.7
Aerobic As(III)	2.4	0.7	3.7	13.6	3.7
Anaerobic As(III)	2.2	0.6	2.5	10.6	4.2
Aerobic As(V)	2.0	0.4	1.0	6.0	6.0
Anaerobic As(V)	1.8	0.5	1.4	7.2	5.1

## Discussion

Although *B. cereus* is a well-known facultative anaerobe, anaerobic respiration of As^5+^ in this group has never been documented. This is the first report on detailed taxonomic analysis of strain IIIJ3-1, phylogenetically belonging to *B. cereus sensu lato* group*,* isolated from As contaminated aquifers of Jorhat, showing remarkable metabolic and genomic potential to respire and resist As. Despite highest sequence similarity of strain IIIJ3-1 with *B. cereus* ATCC 14579(T), neighbor joining tree clearly delineated strain IIIJ3-1 to be a different taxon, phylogenetically distinct from other related members (Fig. 1a). Phylogenetic trees (NJ) (Fig. 1b) constructed with strain IIIJ3-1 and other *Bacillus* spp. resistant to As revealed a close taxonomic relatedness of strain IIIJ3-1 with *B. cereus* AG-27 (AY970345.1), an As resistant strain isolated from a thermal power plant soil at Kanpur (India) [51] with a bootstrap value (100 %). Other As resistant strains *B. indicus* and *B. arsenicus* isolated from As contaminated aquifers of BDP [36, 37], *B. selenatiarsenatis*, *B. arseniciselenatis* strains known for their ability to respire Se^6+^ as well as As^5+^ [46, 47], *B. beveredgei,* a facultative anaerobic haloalkaliphilic strain capable of As respiration [55], *B. foraminis,* isolated from a non-saline alkaline groundwater [56] and *Bacillus* sp. S1 (KF857225), an As resistant strain isolated from As contaminated aquifer of Jorhat (BRB) (data retrieved from NCBI) were found to be phylogenetically distant from strain IIIJ3-1. The phylogenetic analyses indicate that strain IIIJ3-1 is taxonomically distantly related to the previously reported members of *B. cereus* as well as other As resistant members of *Bacillus* sp*.* isolated from elsewhere sites. Interestingly, it is even found to be distantly related to the *Bacillus* sp. reported from Jorhat, similar region from where strain IIIJ3-1 has been isolated (Fig. 1b). Further genomic characterization of strain IIIJ3-1 for DNA-DNA relatedness with the closest NCBI match revealed a ΔT_m_ of 8 °C (Supplementary Fig. S1) which remains well above the suggested limit of 5 ^o^C for species delineation [57] and a characteristic low G+C content corroborated its affiliation to *Bacillus cereus* group.

Chemotaxonomic characterization through bacterial FAME and respiratory quinone analyses also indicated characteristic features of *B. cereus* group along with significant distinct features. Comparison of the fatty acid profile of strain IIIJ3-1 with its closest phylogenetic neighbors revealed prominent differences in its fatty acid composition (Table 3). Like all other members of *B. cereus* group*,* strain IIIJ3-1 is characterized by two fold abundant iC15:0 than aC15:0 and abundance of iC17:0 [58]. Similar to ATCC 14579 (T), presence of C18:1 in strain IIIJ3-1 was also noted [54]. However, presence of hydroxyl fatty acids (HFAs), C11:0 and C18:2^9,12^ with simultaneous absence of iC13:0, iC14:0, iC16:2, aC13:0, aC17:0, aC17:1, nC16:2 and nC17:1 in strain IIIJ3-1 could be considered as distinct chemotaxonomic properties supporting its taxonomic uniqueness from its closest taxonomic relatives.

**Table 3:** Fatty acid composition of *Bacillus cereus* strain IIIJ3-1 and taxonomically related *Bacillus* species

	1	2	3	4	5	6	7	8	9	10	11	12
**Hydroxy- fatty acids**												
2-OH C10:0	**+**	**–**	–	–	–	–	–	–	–	–	–	–
2-OH C12:0	**–**	**–**	–	–	–	–	–	–	–	–	–	–
3-OH C12:0	**+**	**–**	**–**	**–**	**–**	**–**	**–**	**–**	**–**	**–**	**–**	–
2-OH C14:0	**+**	**–**	–	–	–	–	–	–	–	–	–	–
3-OH C14:0	**+**	**–**	–	–	–	–	–	–	–	–	–	–
2-OHC15:0	**–**	**–**	–	–	–	–	+	+	–	–	–	–
2-OH C16:0	**+**	**–**	–	–	–	–	–	–	–	–	–	–
**Branched chain iso fatty acids**												
iC12:0	**–**	**+**	–	–	–	–	–	–	–	+	+	+
iC13:0	**–**	**+**	+	+	+	+	+	+	+	+	+	+
iC14:0	**–**	+	–	–	–	+	+	+	+	+	+	+
iC15:0	**+**	+	+	+	+	+	+	+	+	+	+	+
iC16:0	**+**	**–**	–	–	–	+	+	+	+	+	+	+
iC17:0	**+**	**–**	+	+	+	+	+	+	–	+	–	+
**Branched chain anteiso fatty acids**												
aC13:0	–	+	+	+	+	–	+	+	+	+	+	+
aC15:0	+	+	+	+	+	+	+	+	+	+	+	+
aC17:0	–	+	+	+	+	–	+	+	+	+	+	+
aC17:1	–	+	+	+	+	+	+	+	+	+	+	+
**Saturated fatty acids (SFAs)**												
C11:0	**+**	**–**	**–**	**–**	**–**	**–**	**–**	**–**	**–**	**–**	**–**	**–**
C12:0	**+**	**+**	–	–	–	–	–	+	+	+	+	+
C14:0	**+**	+	+	+	+	+	+	+	+	+	+	+
C15:0	**+**	–	–	–	–	–	–	–	–	–	–	f
C16:0	**+**	+	+	+	+	+	+	+	+	+	+	+
C17:0	**+**	+	–	–	–	–	–	–	–	–	–	f
C18:0	**–**	+	–	–	–	–	–	+	+	+	+	+
C19:0	**+**	–	–	–	–	–	–	–	–	–	–	–
**Unsaturated fatty acids (UFAs)**												
C18:1	**+**	+	–	–	–	–	–	–	–	–	–	f
C16:1ω7cOH	**–**	nr	–	–	–	–	+	–	+	–	+	–
C16:1ω11c	**–**	f	–	–	–	–	–	–	+	–	+	–
nC16:1	**–**	+	–	–	–	–	–	–	–	–	–	+
iC16:2	**–**	+	**–**	**–**	**–**	**–**	**–**	**–**	**–**	**–**	**–**	**–**
nC16:2	**–**	+	**–**	**–**	**–**	**–**	**–**	**–**	**–**	**–**	**–**	**–**
nC17:1	**–**	+	**–**	**–**	**–**	**–**	**–**	**–**	**–**	**–**	**–**	**–**
C18:2^9,12^	**+**	–	–	–	–	–	–	–	–	–	–	–

Strains 1*.* IIIJ3–1 (present study); 2. *B. cereus* ATCC 14579 [54]; 3. B-17, 4. B-19, 5. B-82,(3,4,5) – [58, 59]; 6. *B. toyonensis* BCT-7112^T^; 7, *B. cereus* CECT 148^T^; 8. *B. thuringiensis* CECT 197^T^; 9. *B. mycoides* CECT 4128^T^; 10. *B. pseudomycoides* CECT 7065^T^;; 11. *B. weihenstephanensis* LMG 18989^T^;12. *B. cytotoxicus)* (6–12) [60]. (+ : presence, −:  absence and f:  faint peak for FAME obtained, nr:  no report)

Study on respiratory menaquinone also supported its relatedness to the members of *B. cereus* group with simultaneous unique properties. The molecular identity of quinones provided a further clue to its novelty. While the presence of MK-7 could act as supporting feature for its chemotaxonomic affiliation to *B. cereus* group, presence and abundance of derivatives of MK-5 and MK-6, which has never been reported for members of *B. cereus* group differentiates it from the other members of this group.

**Fig. 6:**
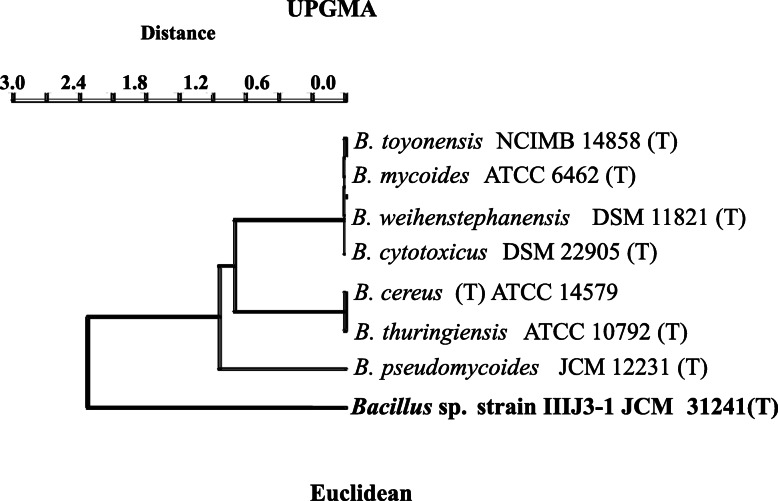
UPGMA analysis for strain IIIJ3–1and its phylogenetic neighbors (members of *B. cereus* group) considering their biochemical and chemotaxonomic traits

Strain IIIJ3-1 is found to share a number of phenotypic and metabolic traits with *B. cereus* group. The physicochemical growth characteristics of strain IIIJ3-1 was found to be consistent with those of other members of genus *Bacillus*. The rhizoidal shape in ageing colonies as found for strain IIIJ3-1 has only been found in *B. mycoides* and *B. pseudomycoides* [52]. Ability to hydrolyze starch and utilize N-acetyl glucosamine, sucrose, trehalose and inability to assimilate mannitol, glycerol, galactose, β-gentiobiose and turanose aligns with the previous findings [36, 37, 46, 52, 53, 60]. However, inability to utilize salicin is a distinct metabolic character of strain IIIJ3-1. Anaerobic growth utilizing alternate TEAs are also in accordance with the previous reports for other members of *Bacillus* [36, 46, 52]. It was suggested that dissimilatory reduction of some metals or metalloids may be a specific character of spore-forming Gram-positive bacteria [61]. Recent geomicrobiological studies have also reported the versatile ability of As-rich groundwater bacteria (*Rhizobium* spp., *Pseudoxanthomonas* sp., *Achromobacter* sp., *Escherichia* spp.) from Bengal basin, India to couple electrons from hydrocarbons and organic acids (lactate, acetate, pyruvate, etc.) to multiple electron acceptors (TEAs) under anaerobic conditions, thus driving the reductive metabolism [16, 62, 63]. Alluvial aquifer of BRB is characterized to be oligotrophic, anoxic, and with fluctuating availability of redox equivalents, lowered derived carbon pool [64]. Considering the overall hydrogeochemistry of BRB groundwater, the metabolic versatility of the strain IIIJ3-1 seems highly justified for its competitive niche adaptation to cope up with fluctuating groundwater condition. Statistical analysis (UPGMA) based on overall chemotaxonomic and physiological properties of strain IIIJ3-1 with other 7 members of *B. cereus* group (Tables 1 and 3) clearly indicated the distinctiveness of this strain from its nearest taxonomic neighbors (Fig. 6). Linear regression and correlation analysis of growth vs coupled reduction profile with stoichiometric imbalance indicated the cellular As uptake during reduction by IIIJ3-1 and its cellular compartmentalization. The observed phenomena corroborated earlier reports on cellular and extracellular adsorption of As during growth by some As-transforming bacterial members from diverse habitat [35, 65–67].

A comparative analysis of kinetic parameters reveals that K_m_ for As^3+^ oxidation (by oxidase) of strain IIIJ3-1 is about 5 times higher than that of *Pseudomonas arsenoxydans* but comparable to that of *Alcaligenes* strain (Supplementary Table 2). K_m_ for As^5+^ reduction (by reductase) was comparable to that of *E.coli* plasmid R773 bearing *ars*C gene and is much lower than that of *S. cereviseae* having Acr2p, but 1-3 folds higher than *Synechocystis* sp. strain PCC 6803*, Pseudomonas stutzeri* and *Staphylococcus aureus* possessing *ars*C genotype [68–70] (Supplementary Table 2). Relatively higher K_m_ towardsAs^5+^ reduction could be attributed to the nonspecific reduction by the cell and this perhaps corroborate well with absence of *ars*C gene encoding cytosolic As^5+^ specific reductase (discussed later).

Preference of lactate over other C-sources by strain IIIJ3-1 under anaerobic growth is in well accordance with the free energy values reported in the literature, where lactate has proved to be the highest energy yielding electron donor with diverse TEAs [68, 71]. Lactate is the readily available fermentation product by many of the naturally occurring bacteria possibly present in such aquifer systems. Therefore, capability to utilize lactate as a C-source by the As^5+^ reducing strain IIIJ3-1 might help in competitive nutrient acquisition and niche adaptation strategy over other bacteria unable to metabolize lactate. Dissimilatory As^5+^ reducing prokaryotes belonging to various genera such as *Desulfitobacterium*, *Desulfuromonas*, *Desulfotomaculum, Sulfurospirillum,* and *Bacillus* capable of utilizing lactate have been reported for their ability to metabolize various other inorganic respiratory substrates as well [32, 46, 47, 72–74]. However, a distinct preference of As^5+^ over other respiratory substrates is a novel characteristic found in strain IIIJ3-1.

Reduction of SO_4_^2-^_,_ NO_3_^-^ and Fe^3+^ was relatively incomplete and their transformation rates varied considerably. Both As^5+^ and NO_3_^-^ revealed similar patterns of reduction and interestingly after reaching the saturation level of reduction (i.e after 15 hours), oxidized substrates were detected in the aqueous phase indicating possible oxidation of the reduced products formed. The observed redox transformation might imply presence of an oxidoreductase system regulating As^5+^ and NO_3_^-^ reduction which may act reversibly when the reduced species reached a threshold concentration. Involvement of a common, non-specific or constitutive oxidoreductase system in *Sulfurospirillum barnesii* SES-3 has been reported earlier [73]. The energetics reported in various studies for lactate oxidation with As^5+^, SO_4_^2-^ and NO_3_^-^ has been tabulated in Supplementary Table 3 [46, 72]. Since, ∆*G*_*f*_° denotes the free energy difference in reactants and the products, lower ∆*G*_*f*_° value indicates higher amount of available energy for cell mass formation. Although NO_3_^-^ was identified as a preferred substrate thermodynamically over As^5+^ and SO_4_^2-^, the present observation on preference of As^5+^ by strain IIIJ3-1 could be attributed to the natural adaptation acquired from its provenance to a highly As contaminated aquifer which might have assisted in the evolution of such physiological characteristic. Relatively lower cell density with As^5+^ compared to NO_3_^-^was reported due to the toxic effect of accumulated As^3+^ formed while growing on As^5+^ [68]. Noticeably, such phenomenon was not evident with strain IIIJ3-1, indicating presence of strong As^3+^ detoxification system operating in the strain while it is growing on As^5+^. Presence of As^3+^ oxidase gene and its activity as present in the strain corroborated such interpretation.

Absence of gene encoding cytosolic As^5+^ reductase (*arsC*) in strain IIIJ3-1 was noted and this observation (presence of *ars*B but absence of arsC) corroborated with a similar finding in *Exiguobacterium* strain WK6 [74]. However, cytosolic As^5+^ reductase activity, very high Km for cytosolic As^5+^ reduction and absence of *ars*C gene suggested some *ars*C independent pathway present in strain IIIJ3-1 for cytosolic As^5+^ reduction. Despite the clear evidence for aerobic As^5+^ reduction by strain IIIJ3-1, an amplicon for As^5+^ reductase (*ars*C) could not be obtained. It might be due to high sequence diversification of *ars*C that the conventional primers for the gene could not target the one present in IIIJ3-1. The As^5+^ respiratory reductase (*arr*) and As^3+^ transporter genes *ars*B and *acr*3(1) appear to be vertically transferred maintaining their phylogenetic consistency. The phylogenetic discrepencies in case of *aio*B suggests possible horizontal transfer of this gene. Strain IIIJ3-1 cells showed alterations in cell morphology and indicated their ability to accumulate As forming electron opaque dots. The As accumulating As^3+^ stressed cells revealed lowering of surface area: volume ratio as a protective mechanism against toxicity of As so as to lower the available attachment/ uptake sites in effect [75, 76]. Relative decrease in cell surface to volume ratio plays the key role in the consequent reduction in attachment/uptake sites on the cell surface for the heavy metals like As in the case of strain IIIJ3-1 [76, 77]. Such stress responses upon exposure to toxic metals, metalloids and organics, other unfavourable conditions like highly acidic or alkaline pH, suboptimal temperatures, is believed to protect vital processes and restore cellular homeostasis, as well as help to enhance cellular resistance against subsequent stress challenges [75, 78, 79]. However, relative decrease in cellular dimensions but, increase in surface area: volume ration in As^5+^ stressed cells suggest an increase in exposure of various other pores or attachment sites to enable the cellular uptake of essential nutrients for cell survival. Cell constriction and appearance of rough cell surface might alter the cellular uptake sites. This might be suggestive of another resistance mechanism towards As stress. Expression of RND proteins (resistance, nodulation and division) for cell survival under stressed condition has been reported for gram negative bacteria [75, 80]. Arsenic accumulation studied through electron microscopy and EDX analysis within microbial cells belonging to *Firmicutes, Actinobacteria* and *Proteobacteria* have been reported in several studies previously [43, 66, 69, 81, 82]. Heavy metal (As, Cd, Zn, Hg, Pb) stress induced changes in cellular morphology have been noticed for many heterotrophic bacterial taxa viz. *Acidocella* sp. GS19h [83], *Acidiphilium symbioticum* H8 [75], *Bacillus* members (*B. arsenicus*, *B. pumilus*, *B. indicus*) [76] and *Bacillus* sp. strain XZM002 [43]. Substantial reduction in cell sizes has been ascribed to protective mechanism of the bacterial strain to cope up with metal toxicity. Overexpression of a metalloregulatory protein ArsR with high affinity for As^3+^ in *E.coli* showed increased bioaccumulation which allowed 100% removal of 5- ppb of As^3+^ from water [84], Another bacterium *Pseudomonas stutzeri* could accumulate As upto4 mg/gram of biomass (dry weight) [85]. Intracellular As accumulation has also been confirmed through TEM and EDAX analysis in isolates belonging to *γ-Proteobacteria*, *Firmicutes* and *Kocuria* [66]. However, none of the isolates have been shown to accumulate As during their anaerobic growth with As^3+^.Formation of small spheres composed of Se and encapsulated with polymeric material on the periphery of the cell envelope of *B. selenitireducens* on growth with Se^4+^ has been reported [46]. However, formation of electron opaque dots specifically following growth on the reduced species of As i.e. As^3+^ by strain IIIJ3-1 is a characteristic never reported earlier for any other bacteria. This could possibly indicate a novel mechanism to sustain the toxic effects of As^3+^ wherein, the bacteria complexes the inorganic ion in immobilized particles thus making it unavailable to the cellular machinery. It might also reflect that the cells upon As^3+^ oxidation are incapable of extruding As^5+^ and thus accumulate As within, whereas, while growing with As^5+^, As^3+^ formed from its reduction could be easily extruded out of the cell. Presence of As^3+^ extruding genes further supports this observation. Since the *aio* positive and *arsC* negative IIIJ3-1 bacterial cells were found to accumulate As only under As^3+^ stress, it proves that As^3+^ upon oxidation to As^5+^ cannot be further extruded out of the cell due to the lack of respective cellular machinery as also reported by Joshi et al. [86]. Moreover, As biotransformation assay for strain IIIJ3-1 has also shown very high K_m_ for cytosolic As reduction implying presence of some non-specific reductase. This is also explained by Yang et al., [87] in *Sinorhizobium meliloti*, As^5+^ is reduced to As^3+^ via *arsC*, and AqpS confers resistance by downhill efflux of internally generated As^3+^. But in case of IIIJ3-1, the reduction of cytosolic As^5+^ does not occur/ or occur non-specifically due to absence of *arsC* gene. However, the threshold of As^5+^ accumulation has not been calculated and is an important aspect to be considered for our future scope of research. Several bacterial members have been known to accumulate As within their cellular components during growth under As^5+^ reducing and As^3+^ oxidizing condition upto varying extents. When incubated with 5 mg/L of As^5+^*Marinomonas communis* accumulated 2290 μg As/g dry weight, the highest value reported in bacteria [88]. Many other bacterial members reported to accumulate 110–765 μgAs/g dry wt. [84, 89, 90]. The highest amount of As accumulation reported is 765 μg/g dry wt. in *E. coli* without ars operon and this was ascribed to the inefficiency of this bacterium to extrude As out of cell during growth [84]. In one of the *Bacillus* sp. strain DJ-1, the lack of *ars*C gene and arsenate reductase activity was noted with 80% of As accumulation in cytoplasm, where a DNA protection during starvation (DPS) protein was reported to involve in As-binding in the cytoplasm to reduce the intracellular As toxicity [85]. It has been shown that for *Marinomonas communis*, ~45 % of total As was incorporated into the cytosol, 10 % lipid-bound in the membrane, and rest 45 % adsorbed onto the cell surface [88]. Although the details of the compartmentalization has not been studied in detail, the accumulation of As has been ascribed to be the result of higher uptake and lower efflux by the bacterium. Similarly approximately 80 % of the total accumulated As (11.5 % of total) was adsorbed onto the membrane instead of into cytoplasm of *Bacillus* sp. XZM002 from As-rich Datong basin, China [91]. However, EDX analysis shows maximum content of As under anaerobic growth with As^3+^. This might indicate that cells grown anaerobically with As^3+^ might have utilised As^3+^ as TEA as reduction of As^3+^ to As^0^ or As^3-^ has been reported to be energetically favourable [68].

## Conclusion

Arsenic contamination in the Brahmaputra river basin is a natural calamity which has degraded the potability of groundwater of various areas in this region. Very few reports are available to understand the microbiology of these aquifers wherein, recently Das and Barooah [38] have documented the siderophore based role of an As resistant *Staphylococcus* strain TA6 in As mobilization in these valleys. However, immediate concern is imperative to remediate the groundwater of these areas to restore its potability. This study presents a detailed molecular, chemotaxonomic, biochemical and ecophysiological characterization of a novel member of *B. cereus* strain IIIJ3-1 capable of efficiently transforming and accumulating As^3+^. Evidently, based on its chemotaxonomic, genomic and metabolic properties, strain IIIJ3-1 represents a novel and non-clonal member of *B. cereus* group which can act as potential As^3+^ accumulator in As contaminated regions. Based on its capability to survive and accumulate the most toxic form of inorganic As (As^3+^) within its system, the bacterial strain IIIJ3-1 offers a novel mechanism of As^3+^ remediation in As- contaminated groundwater as well as heavily contaminated irrigational fields as found in West Bengal [92]. Further investigation is called for standardizing and designing of bioremedial procedures using this highly efficient As biosequestering strain *Bacillus* IIIJ3-1.

## Methods

### Bacterial strains and growth condition

Strain IIIJ3-1 (JCM 31241(T)) was previously isolated [93], sub-cultured and routinely maintained on Luria Bertani (LB) agar or Reasoner’s 2A (R2A) agar, unless otherwise indicated. Type strain *B. cereus* ATCC 14579 was obtained from ATCC, cultured on appropriate medium, and used for comparison of phenotypic, chemotaxonomic and genotypic characteristics.

### 16S rRNA gene sequencing and phylogenetic analysis of strain IIIJ3–1

The genomic DNA of strain IIIJ3–1 was extracted using Pure Link genomic DNA kit (Invitrogen). The 16S rRNA gene was amplified by PCR with bacterial universal primers (27Fand1492R) following PCR conditions as mentioned in [39]. The PCR master mix was prepared according to the manufacturer’s protocol (NEB). The PCR cycle composed of an initial denaturation at 95 °C for 5 min followed by 30 cycles of denaturation at 95 °C for 1 min, annealing at 58 °C for 1.5 min, an extension at 72 °C for 50 s and a final extension at 72 °C for 1 min. The PCR products were gel purified using a Qia-quick gel extraction kit (QIAGEN), cloned into pTZ57R/T vector (InsTA clone kit, Thermo scientific), and sequenced using internal primers: 27F, 341F, 811F and 1492R to obtain near complete sequence of 16S rRNA. Homology search for maximum similarity of the 16S rRNA gene sequence of strain IIIJ3–1 was carried out using identity tool of EzTaxon-e server (https://www.ezbiocloud.net/identify) and NCBI database. The Ez Taxon showed % similarity with reported type strains and similarity with type and non-type strains were obtained from NCBI database. Multiple alignments were performed with CLUSTALW package of MEGA 7.0 software [94] by removing gaps from the sequences. Phylogenetic reconstruction and validation were performed using neighbor-joining (NJ) method [95] based on bootstrap analysis with 1000 replications using Jukes-Cantor [96] distance model. Both maximum-likelihood (ML) [97] and minimum-evolution (ME) [98] methods were employed to test the robustness of the trees.

### Determination of molar G + C % and DNA-DNA relatedness

Molar G+C content (%) of strain IIIJ3-1was determined using the thermal denaturation method [57, 99, 100] where *Escherichia coli* K-12 NCIM 2563 used as the internal standard. DNA-DNA relatedness of strain IIIJ3-1 with its nearest phylogenetic neighbor *B.cereus* ATCC 14579(T) was measured using the SyBr green binding fluorimetry based method and relative binding ratio (RBR) was calculated following the correlation equation: y = (-)5.051x + 90.329 relating DNA - DNA relatedness and ∆T_m_ [100].

### Chemotaxonomic (fatty acid methyl esters and quinone content) analysis

Total cellular fatty acid methyl ester (FAME) profiles of strain IIIJ3-1 and its closest phylogenetic neighbors (type strains) were determined by the growing cells of respective strains on LB medium for 24h at 30 °C [101]. Cellular fatty acids were saponified, methyl-esterified, and extracted according to the protocol of the Sherlock Microbial Identification System (MIDI). The FAMEs were analyzed using Gas Chromatograph (GC, Clarus 500, PerkinElmer) and compared with standard bacterial acid methyl ester mix (BAME, Sigma) for their identity. Isoprenoid quinines were extracted following the protocol mentioned by Dispirito et al. [102] separated using a SB-C18 Zorbax reverse-phase column fixed to a High Pressure Liquid Chromatograph (1100 Series, Agilent) with a solvent system of methanol: isopropanol (75:25 v/v) maintaining a flow rate of 1ml/min. Fractions corresponding distinct peaks at 2.9, 4.3 and 8.3 minutes were collected and concentrated in an evaporator. Molecular masses of the constituent quinones were analyzed by 4800 MALDI TOF/TOF analyzer (Applied BiosystemsInc., USA) using sinapinic acid as matrix. Constituent menaquinones were analysed by comparing the molecular masses in NCBI PubChem (https://pubchem.ncbi.nlm.nih.gov/).

### Phenotypic and metabolic characterization of strain IIIJ3–1

Culture characteristics of strain IIIJ3-1 were observed by growing cells on LB agar plates for 24 h at 30°C.Cellular morphology was examined by scanning electron microscope (SEM, Zeiss, Evo 60), after harvesting cells from mid-exponential growth phase. For SEM, cells were washed with phosphate buffer (1X), fixed with 0.25% glutaraldehyde (Sigma) and para-formaldehyde (4%, v/v) in PBS at 4 °C, serially dehydrated (30-100% ethanol, v/v), spotted onto poly-L-Lysine coated cover slips, dried, and viewed after gold coating. Motility was determined by the hanging-drop technique [103]. Gram staining was performed using a Gram stain kit according to the manufacturer’s instructions (Hi-Media). Catalase and oxidase tests were performed by testing bubble formation ability of the isolate on treatment with H_2_O_2_ (30%) and using oxidase reagent (Biomerieux) according to the manufacturer’s protocol, respectively. Sensitivity towards range of temperatures (5-50 °C, at the interval of 5 °C), pH (5.0-12.0, at the interval of 1 pH unit), NaCl concentrations (0-8%, at the interval of 0.5 %) was evaluated by growing cells of strain IIIJ3-1 in LB broth for 24-48 h. Cell growth under the varying temperature, pH and osmolarity was monitored by recording the culture absorbance (OD at 600 nm) using a UV-Visible spectrophotometer (Cary 50, Varian). Resistance towards As species (As^3+^ and As^5+^) was tested following growth of cells of strain IIIJ3-1 in LB broth amended with graded concentrations of As^3+^ (NaAsO_2_; 0.1-30 mM) and As^5+^ (Na_2_HAsO_4_; 1-600 mM). The highest concentration of As species, up to which growth was observed was considered as maximum tolerable concentration (MTC). Medium without As was treated as control. Assimilation of broad range carbon and nitrogen substrates, susceptibility towards various antibiotics, and ability to withstand metabolic inhibitors were tested using the Biolog system (GEN-III MicroPlate, Biolog) following the manufacturer’s instructions. Statistical analysis involving the phenotypic and biochemical properties of strain IIIJ3-1 and other *Bacillus* spp. were performed using hierarchical cluster analysis through unweighted paired group arithmetic mean (UPGMA) calculation using Euclidean distance matrix with complete linkage algorithm in Multi-variate statistical package (MVSP) software. Linear regression and correlation analyses involving cellular growth profile and substrate reduction kinetics were performed using Excel 2010 and Minitab Statistical software 17.0.

### Test for arsenic transformation by resting cells of strain IIIJ3–1

#### Arsenic biotransformation assay

Kinetic assay for As^3+^ oxidase and cytosolic As^5+^ reductase activities of the strain IIIJ3-1 was performed under aerobic condition. Cells of strain IIIJ3-1 were grown in LB medium (300 ml) devoid of any As until mid-log phase (O.D_600_= 1.3). The cells were harvested and washed thrice with reaction buffer, 1X TE (Tris,1M; EDTA,100mM), re-suspended in 10 ml of reaction buffer with concentration gradient of As^3+^ or As^5+^(1-5 mM) and allowed to stand for24 h at 30°C. Concentrations of As^3+^ and As^5+^ were measured following molybdenum blue method [104] at regular intervals. All the experiments were repeated thrice with each set in duplicates and mean of all results were used to calculate the kinetic parameters. Kinetic parameters, K_m,_ and V_max_ were calculated by plotting respective Line-Weaver Burk plots.

#### Test for use of different electron acceptors by strain IIIJ3–1

Anaerobic growth with alternate terminal electron acceptors (TEAs) *viz.* As^5+^, Se^6+^, Fe^3+^, S_2_O_3_^2-^, NO_3_^-^_,_and SO_4_^2-^ (1 mM each) in reduced LB medium was monitored by observing absorbance at 600 nm using a UV spectrophotometer (Cary win UV, Agilent Technologies) and colony forming units (CFU) per ml at definite intervals till 54 h. Cysteine-HCl (0.1% as reducing agent) and resazurin (0.1 mg/L as redox indicator) were added to the degassed medium and the vials were crimp sealed. This procedure was followed throughout the work for setting up anaerobic growth for various experiments. Utilization of various electron donors during anaerobic growth with As^5+^as TEA was tested by growing strain IIIJ3-1 in reduced minimal salt medium (MSM) [Composition (g/L): NH_4_Cl (0.535); KH_2_PO_4_ (0.136)_;_ MgCl_2_. 6H_2_O (0.204); CaCl_2_.2H_2_O (0.147); Na_2_MoO_4_ (0.01); Cys-HCl 0.1%, v/v] supplemented with alternate electron donors (10mM). Growth was monitored by measuring the culture turbidity at 660 nm after 15 days. Anaerobic reduction profile of strain IIIJ3-1 with alternate TEAs: As^5+^, SO_4_^2-^ (1 mM) and Fe^3+^, NO_3_^-^ (5 mM) was also studied in MSM with lactate as the sole electron donor by measuring the concentrations of As^5+^, SO_4_^2-^, NO_3_^-^ and Fe^2+^ produced from Fe^3+^ reduction [104–107] at an interval of 3 h. A vial without any electron donor and acceptor was subjected to similar growth conditions which served as the control.

### Scanning electron microscopy (SEM) and energy-dispersive X-ray spectroscopy (EDX)

For visualization of the structural changes of the cells of strain IIIJ3-1 while growing under different As amended conditions (24 hr), scanning electron microscopy (SEM) was performed following the same protocol mentioned before (as described in the phenotypic characterization). In order to confirm the cellular As-uptake and -accumulation, energy dispersive x-ray spectroscopy (EDX) were performed using EDX analyzer (Oxford Instruments) in conjunction with SEM microscope. The morphometric analysis of the strain IIIJ3-1 grown with and without As stress was done by calculating the cell volume (V) and surface area (A) using the following equations:

$$ A=2\pi {r}^2+2\pi rh $$

$$ V=\pi {r}^2h $$

Where, A is the surface area (μm^2^), V is the volume (μm^3^), r is the radius and h is the length of the cell. The average dimensions of the non-dividing cells in SEM image presented have been calculated [108].

### Analysis of functional genes related to as transformation

Genes responsible for As^3+^ oxidation (*aio*B)*,* cytosolic As^5+^ reduction (*ars*C), respiratory As^5+^ reduction (*arr*A)*,* arsenite efflux pumps (*ars*B) and (*acr*3) were targeted for PCR amplification from genome of strain IIIJ3-1 by colony lysis method using reported sets of primers and PCR conditions [13]. The desired amplified fragments were cloned in pTZRT57 (Insta cloning kit, Fermentas), sequenced using M13F and T7R primers, and the obtained nucleotide sequences were searched for similarity level using BLASTN. The corresponding nucleotides were translated to amino acids in ExPasy tool (https://web.expasy.org/translate/) using appropriate open reading frames (ORFs) and searched in BLASTP, (nr database) excluding options for uncultured/environmental sequences. Conserved domain was predicted through CDD database and phylogeny was inferred through Maximum Likelihood tree construction using MEGA 7.0 considering the translated amino acid sequence of strain IIIJ3-1 and similar sequences retrieved from NCBI Multiple alignments of deduced amino acid sequences was done using Clustal Omega and ESPript [109] to decipher the conserved/consensus active site residues for each functional gene.

### Nucleotide sequence accession numbers

The nucleotide sequences of all the functional genes of strain IIIJ3-1 have been submitted to NCBI-Genbank under accession numbers: KJ769230 (16S rRNA gene), and KY024786- KY024788 (As-responsive genes), respectively.

## Supplementary information

**Additional file 1.**

**Additional file 2.**

## Data Availability

The nucleotide sequences of the genes analysed are submitted in the NCBI Genbank. Strain IIIJ3-1 has been submitted to three culture collections- MCC 2980, LMG 29433, JCM 31241.

## Abbreviations

BAME: Bacterial Acid Methyl Ester
BDP: Bengal Delta Plain
BLAST: Basic Local Alignment Search Tool
BRB: Brahmaputra River Basin
CFU: Colony Forming Unit
DARB: Dissimilatory As^5+^ Respiring Bacteria
DPS: DNA Protection during Starvation
EDX: Energy Dispersive X-ray
FAME: Fatty Acid Methyl Ester
GC: Gas Chromatography
LB: Luria Bertani
MALDI TOF-MS: Matrix Assisted Laser Desorption Ionization-Time of Flight Mass Spectrometry
MCC: Microbial Culture Collection
ME: Minimum Evolution
MEGA: Molecular Evolutionary and Genetic Analysis
MK: Menaquinone
ML: Maximum Likelihood
MTC: Maximum Tolerable Concentration
MTCC: Microbial Type Culture Collection
MVSP: Multivariate Statistical Package
NCBI: National Centre for Biotechnology Information
NJ: Neighbour Joining
OD: Optical Density
ORF: Open Reading Frame
PCR: Polymerase Chain Reaction
R2A: Reasoner’s 2A
RBR: Relative Binding Ratio
RND: Resistance, Nodulation and Division
SEM: Scanning Electron Microscopy
TEA: Terminal Electron Acceptor
TEM: Transmission Electron Microscopy
UPGMA: Unweighted Paired Group Arithmetic Mean
