# Supplementary material for: Molecular and taxonomic characterization of arsenic (As) transforming Bacillus sp. strain IIIJ3–1 isolated from As-contaminated groundwater of Brahmaputra river basin, India
Source: BMC Microbiol. 2020 Aug 17;20:256. doi: 10.1186/s12866-020-01893-6 (PMC7430025; doi:10.1186/s12866-020-01893-6)
Supplement: Supplementary file 1 — Additional file 1. [file 12866_2020_1893_MOESM1_ESM.pptx]

## Slide 1
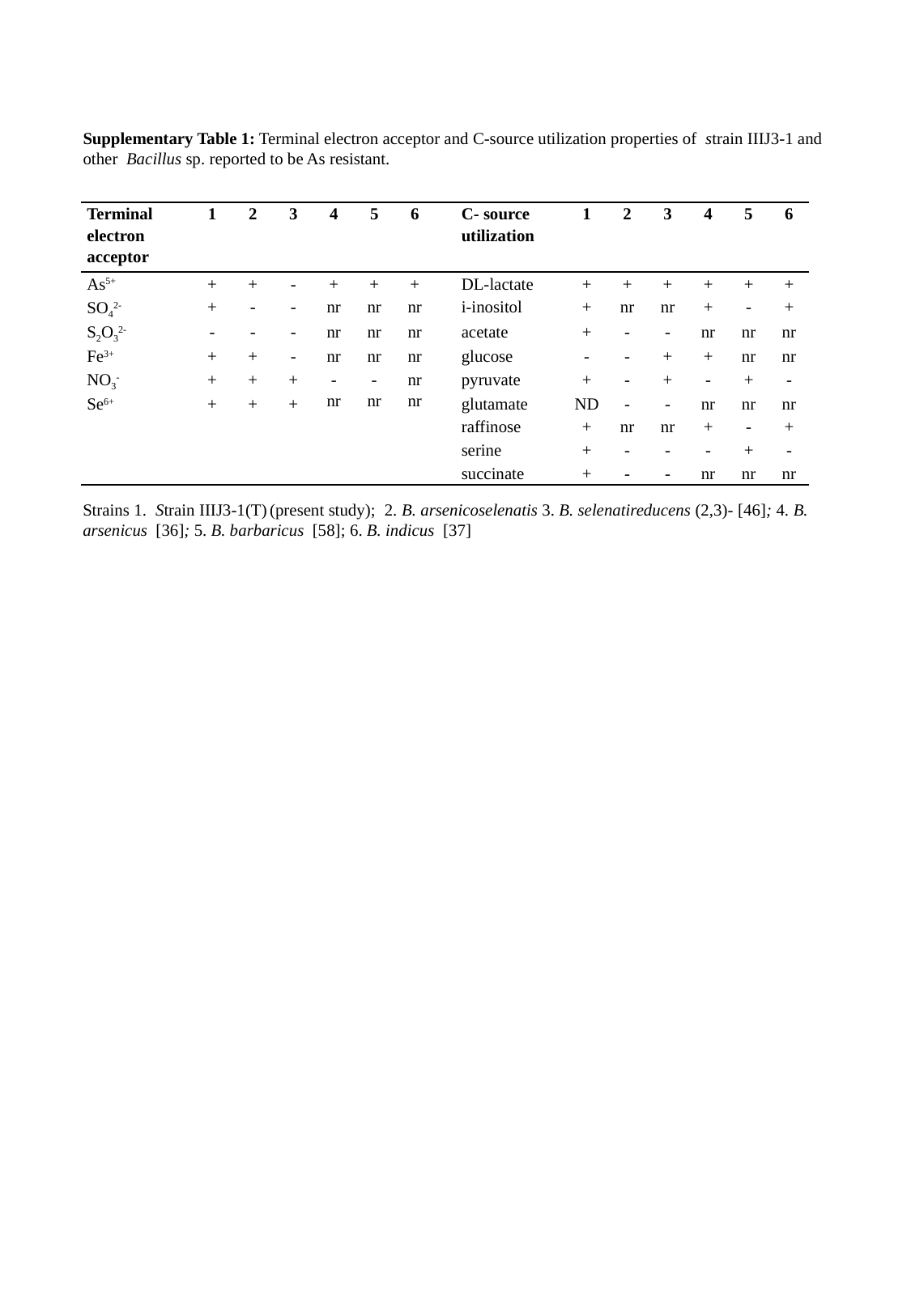

Supplementary Table 1: Terminal electron acceptor and C-source utilization properties of strain IIIJ3-1 and other Bacillus sp. reported to be As resistant.
| Terminal electron acceptor | 1 | 2 | 3 | 4 | 5 | 6 | | C- source utilization | 1 | 2 | 3 | 4 | 5 | 6 |
| --- | --- | --- | --- | --- | --- | --- | --- | --- | --- | --- | --- | --- | --- | --- |
| As5+ | + | + | - | + | + | + | | DL-lactate | + | + | + | + | + | + |
| SO42- | + | - | - | nr | nr | nr | | i-inositol | + | nr | nr | + | - | + |
| S2O32- | - | - | - | nr | nr | nr | | acetate | + | - | - | nr | nr | nr |
| Fe3+ | + | + | - | nr | nr | nr | | glucose | - | - | + | + | nr | nr |
| NO3- | + | + | + | - | - | nr | | pyruvate | + | - | + | - | + | - |
| Se6+ | + | + | + | nr | nr | nr | | glutamate | ND | - | - | nr | nr | nr |
| | | | | | | | | raffinose | + | nr | nr | + | - | + |
| | | | | | | | | serine | + | - | - | - | + | - |
| | | | | | | | | succinate | + | - | - | nr | nr | nr |
Strains 1. Strain IIIJ3-1(T) (present study); 2. B. arsenicoselenatis 3. B. selenatireducens (2,3)- [46]; 4. B. arsenicus [36]; 5. B. barbaricus [58]; 6. B. indicus [37]

## Slide 2
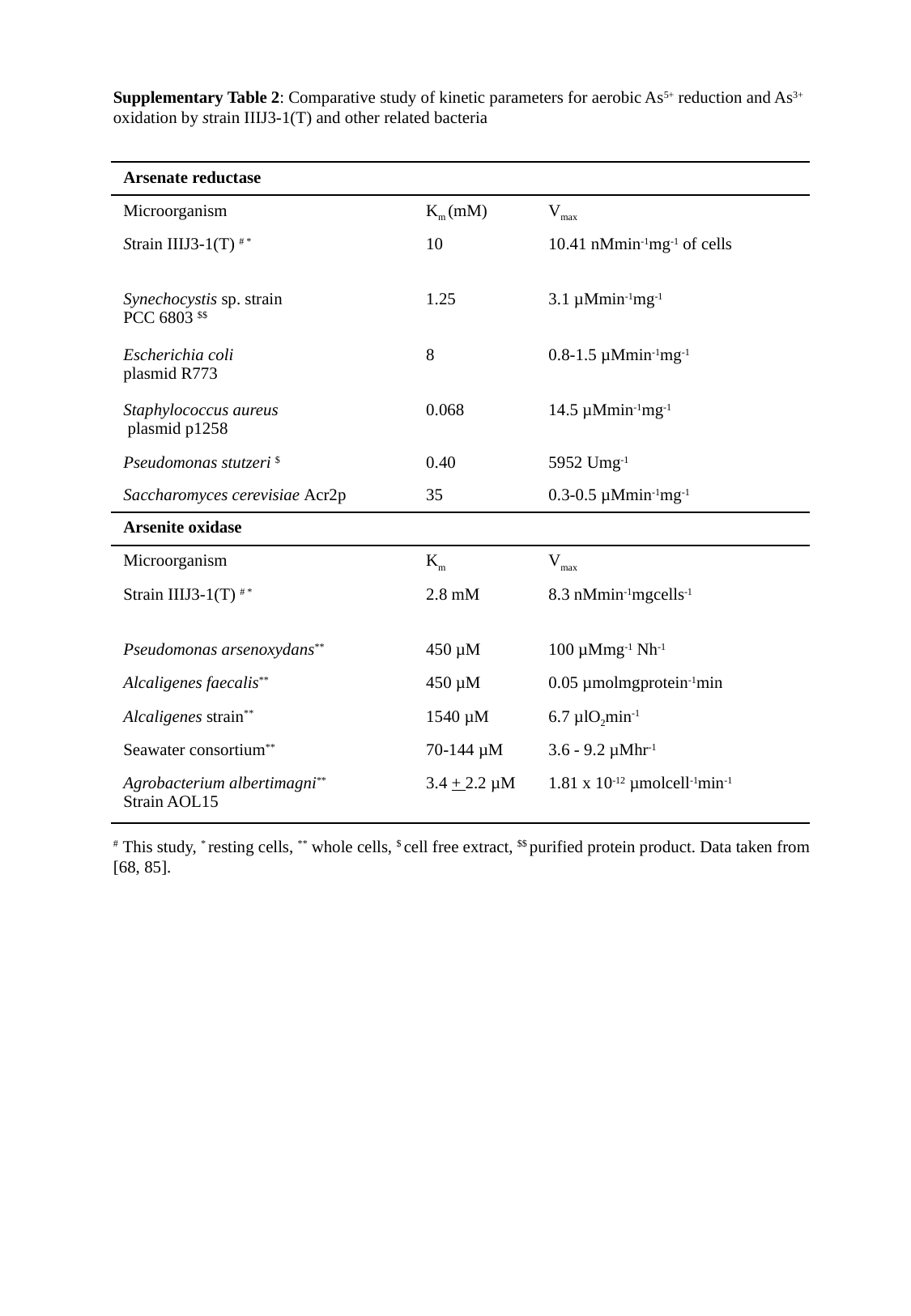

Supplementary Table 2: Comparative study of kinetic parameters for aerobic As5+ reduction and As3+ oxidation by strain IIIJ3-1(T) and other related bacteria
| Arsenate reductase | | |
| --- | --- | --- |
| Microorganism | Km (mM) | Vmax |
| Strain IIIJ3-1(T) # \* | 10 | 10.41 nMmin-1mg-1 of cells |
| Synechocystis sp. strain PCC 6803 $$ | 1.25 | 3.1 µMmin-1mg-1 |
| Escherichia coli plasmid R773 | 8 | 0.8-1.5 µMmin-1mg-1 |
| Staphylococcus aureus plasmid p1258 | 0.068 | 14.5 µMmin-1mg-1 |
| Pseudomonas stutzeri $ | 0.40 | 5952 Umg-1 |
| Saccharomyces cerevisiae Acr2p | 35 | 0.3-0.5 µMmin-1mg-1 |
| Arsenite oxidase | | |
| Microorganism | Km | Vmax |
| Strain IIIJ3-1(T) # \* | 2.8 mM | 8.3 nMmin-1mgcells-1 |
| Pseudomonas arsenoxydans\*\* | 450 µM | 100 µMmg-1 Nh-1 |
| Alcaligenes faecalis\*\* | 450 µM | 0.05 µmolmgprotein-1min |
| Alcaligenes strain\*\* | 1540 µM | 6.7 µlO2min-1 |
| Seawater consortium\*\* | 70-144 µM | 3.6 - 9.2 µMhr-1 |
| Agrobacterium albertimagni\*\* Strain AOL15 | 3.4 + 2.2 µM | 1.81 x 10^-12^ µmolcell-1min-1 |
# This study, * resting cells, ** whole cells, $ cell free extract, $$ purified protein product. Data taken from [68, 85].

## Slide 3
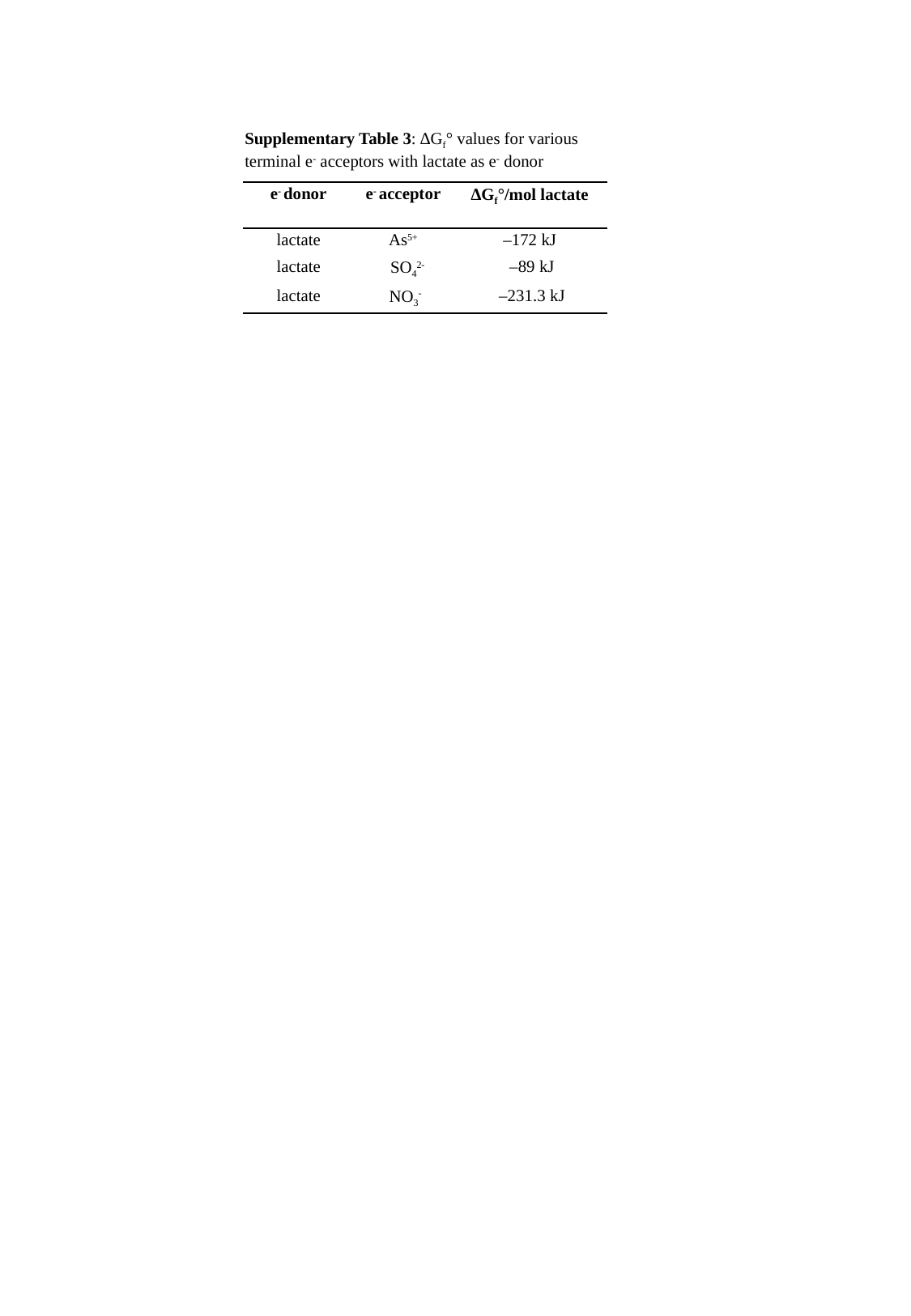

Supplementary Table 3: ∆Gf° values for various terminal e- acceptors with lactate as e- donor
| e- donor | e- acceptor | ∆Gf°/mol lactate |
| --- | --- | --- |
| lactate | As5+ | –172 kJ |
| lactate | SO42- | –89 kJ |
| lactate | NO3- | –231.3 kJ |
