# Supplementary material for: Molecular and taxonomic characterization of arsenic (As) transforming Bacillus sp. strain IIIJ3–1 isolated from As-contaminated groundwater of Brahmaputra river basin, India
Source: BMC Microbiol. 2020 Aug 17;20:256. doi: 10.1186/s12866-020-01893-6 (PMC7430025; doi:10.1186/s12866-020-01893-6)
Supplement: Supplementary file 2 — Additional file 2. [file 12866_2020_1893_MOESM2_ESM.pptx]

## Slide 1
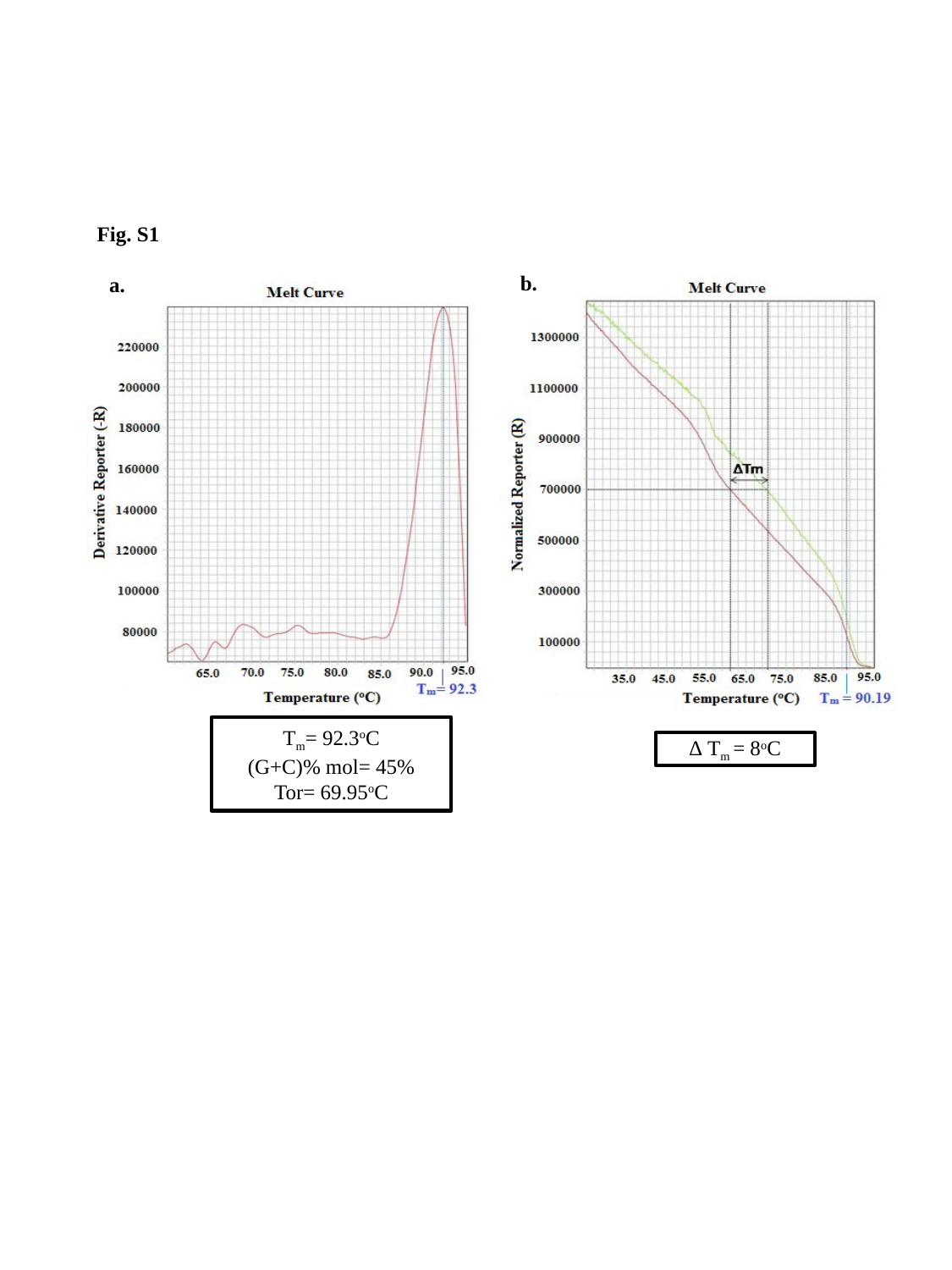

Fig. S1
b.
a.
Tm= 92.3oC
(G+C)% mol= 45%
Tor= 69.95oC
∆ Tm = 8oC

## Slide 2
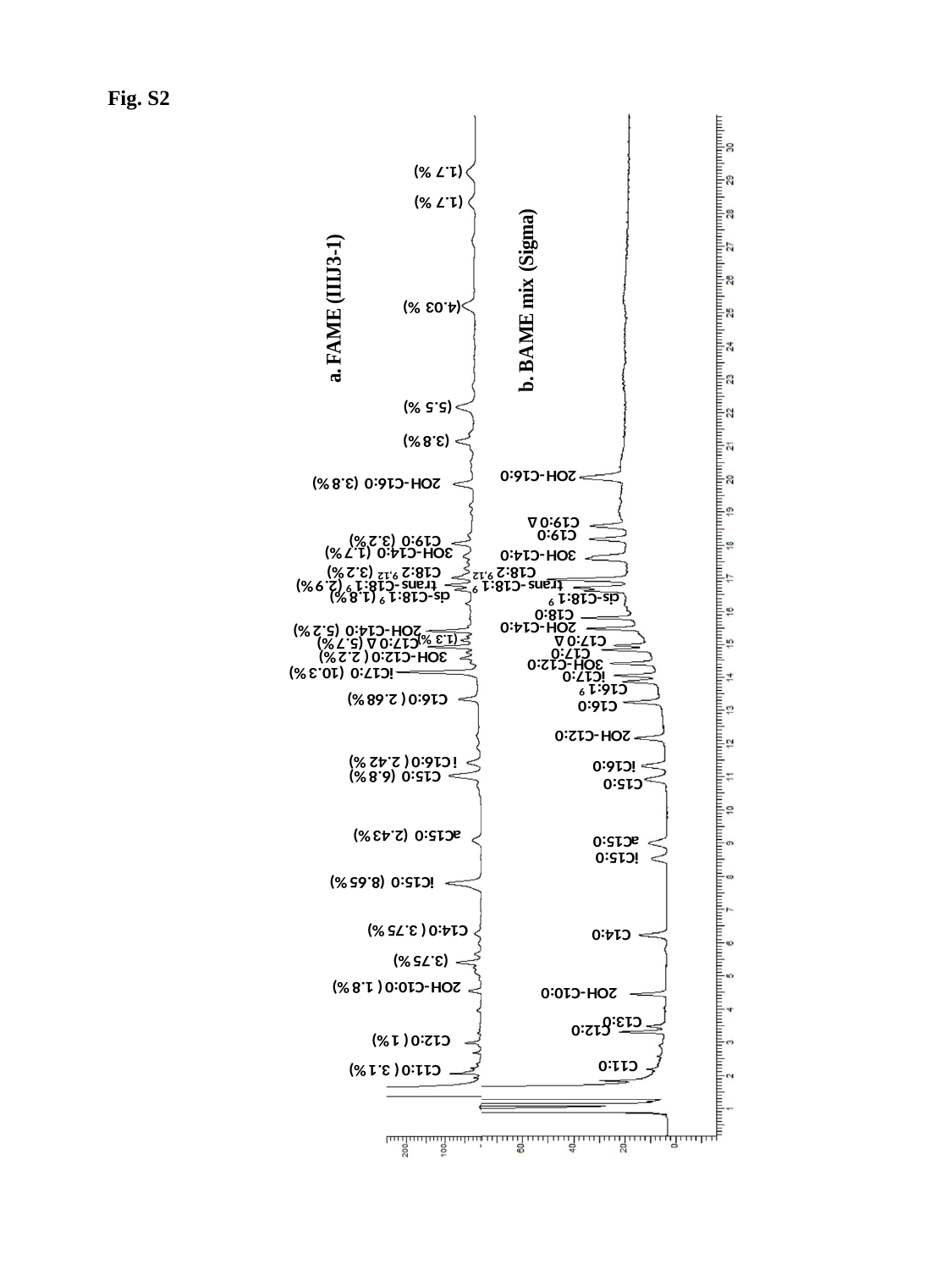

Fig. S2

## Slide 3
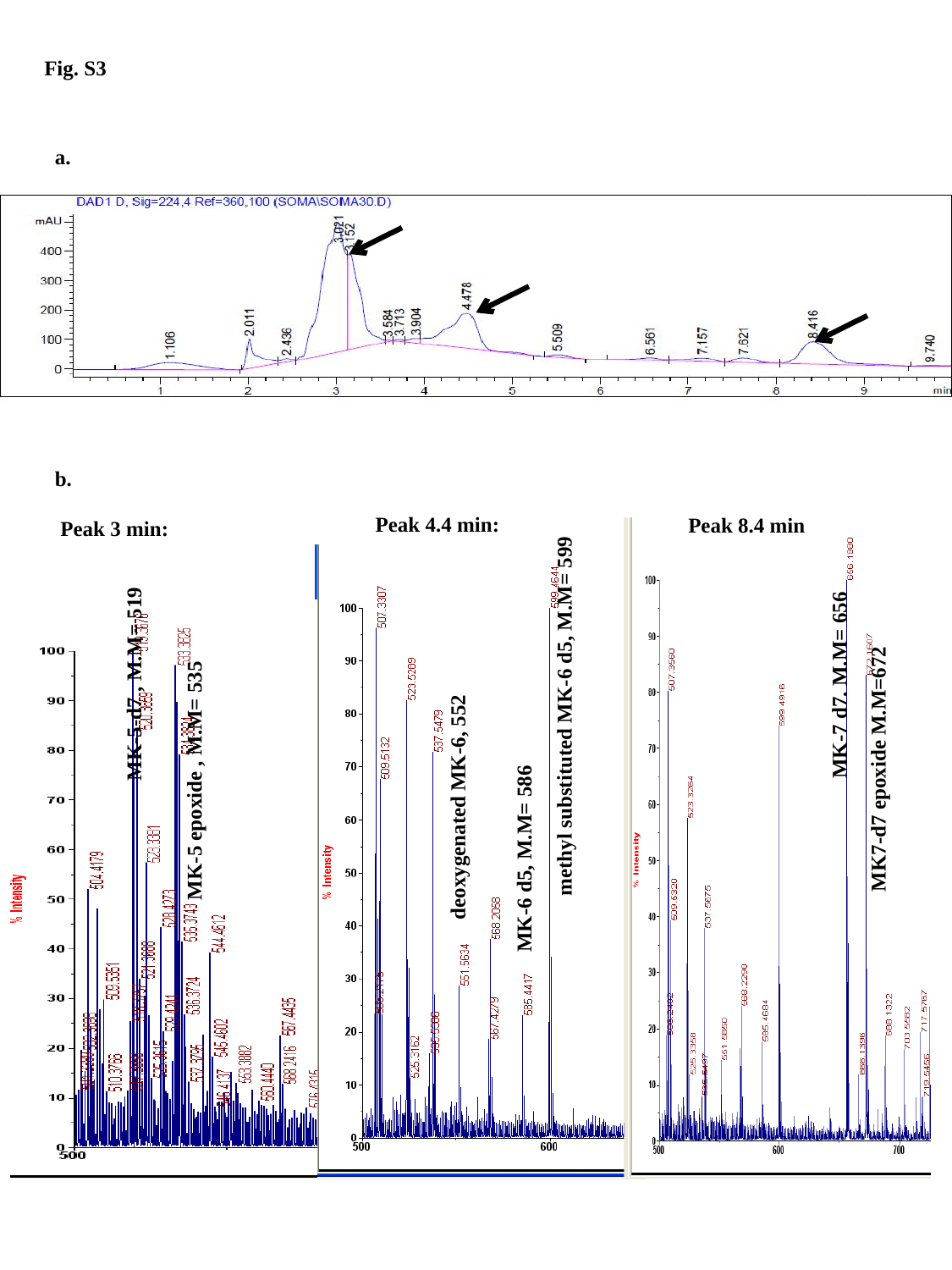

Fig. S3
a.
b.
Peak 8.4 min
MK-7 d7. M.M= 656
MK7-d7 epoxide M.M=672
Peak 4.4 min:
methyl substituted MK-6 d5, M.M= 599
deoxygenated MK-6, 552
MK-6 d5, M.M= 586
Peak 3 min:
MK-5-d7 , M.M= 519
MK-5 epoxide , M.M= 535

## Slide 4
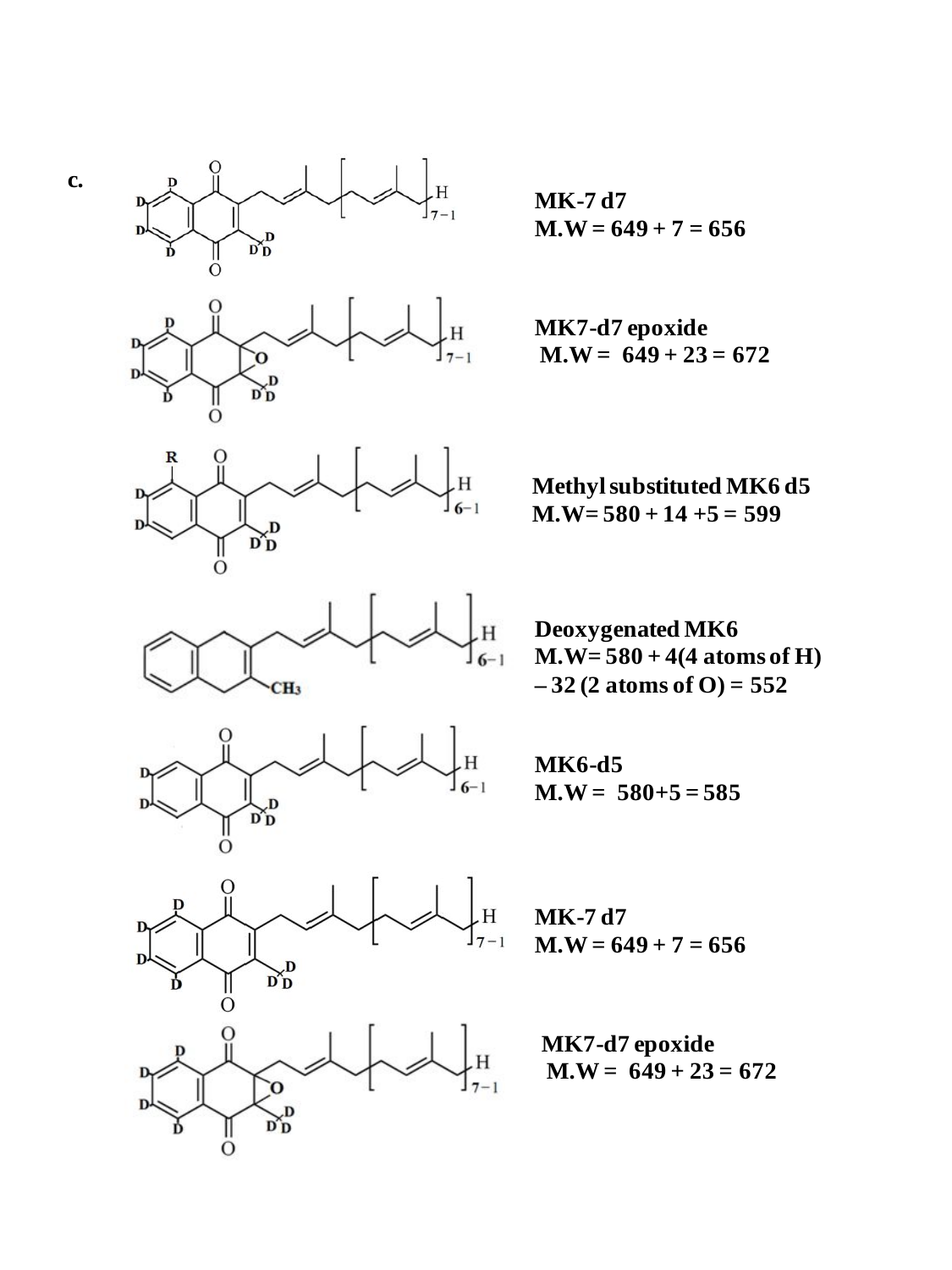

## Slide 5
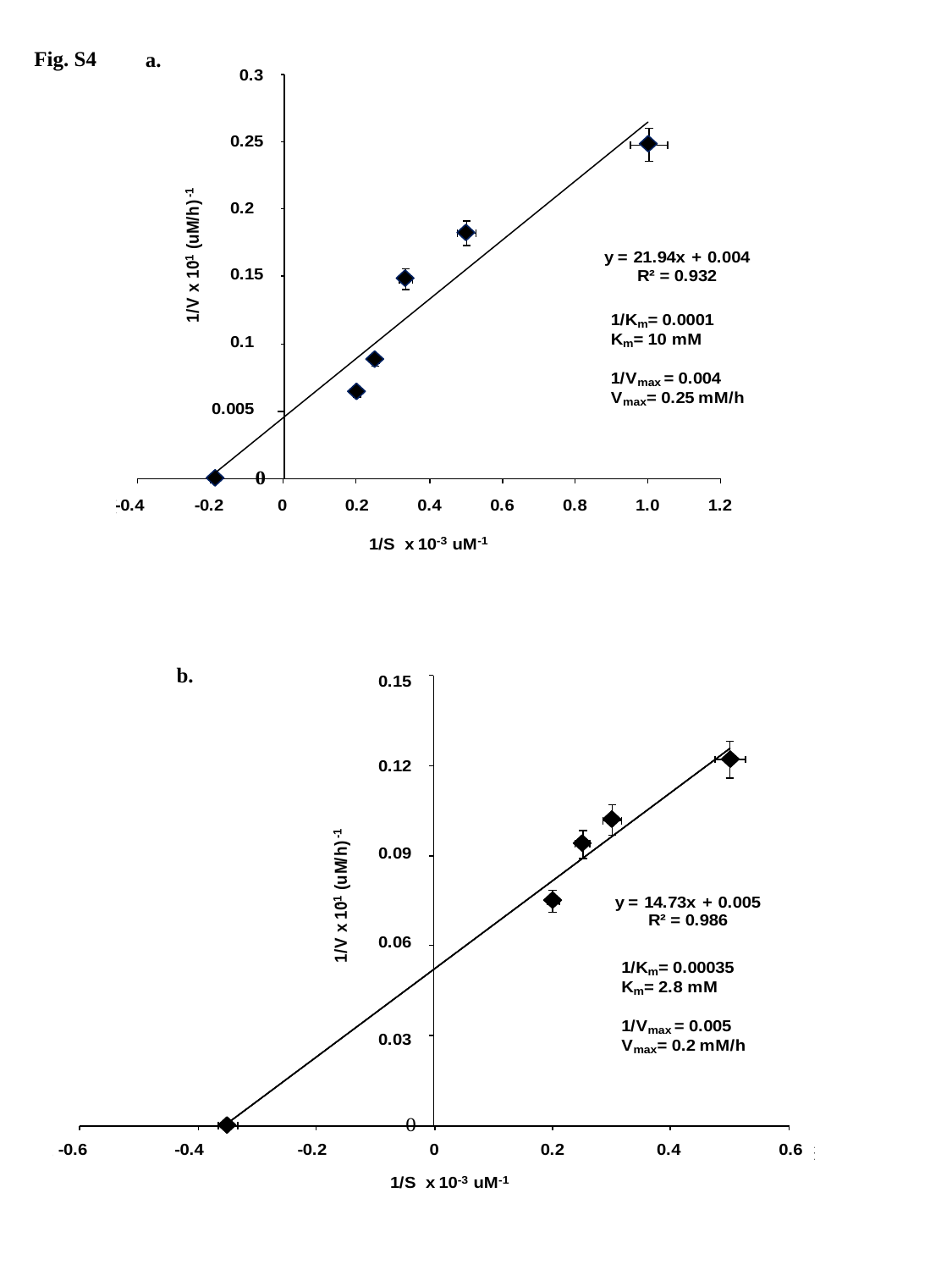

a.
Fig. S4
b.

## Slide 6
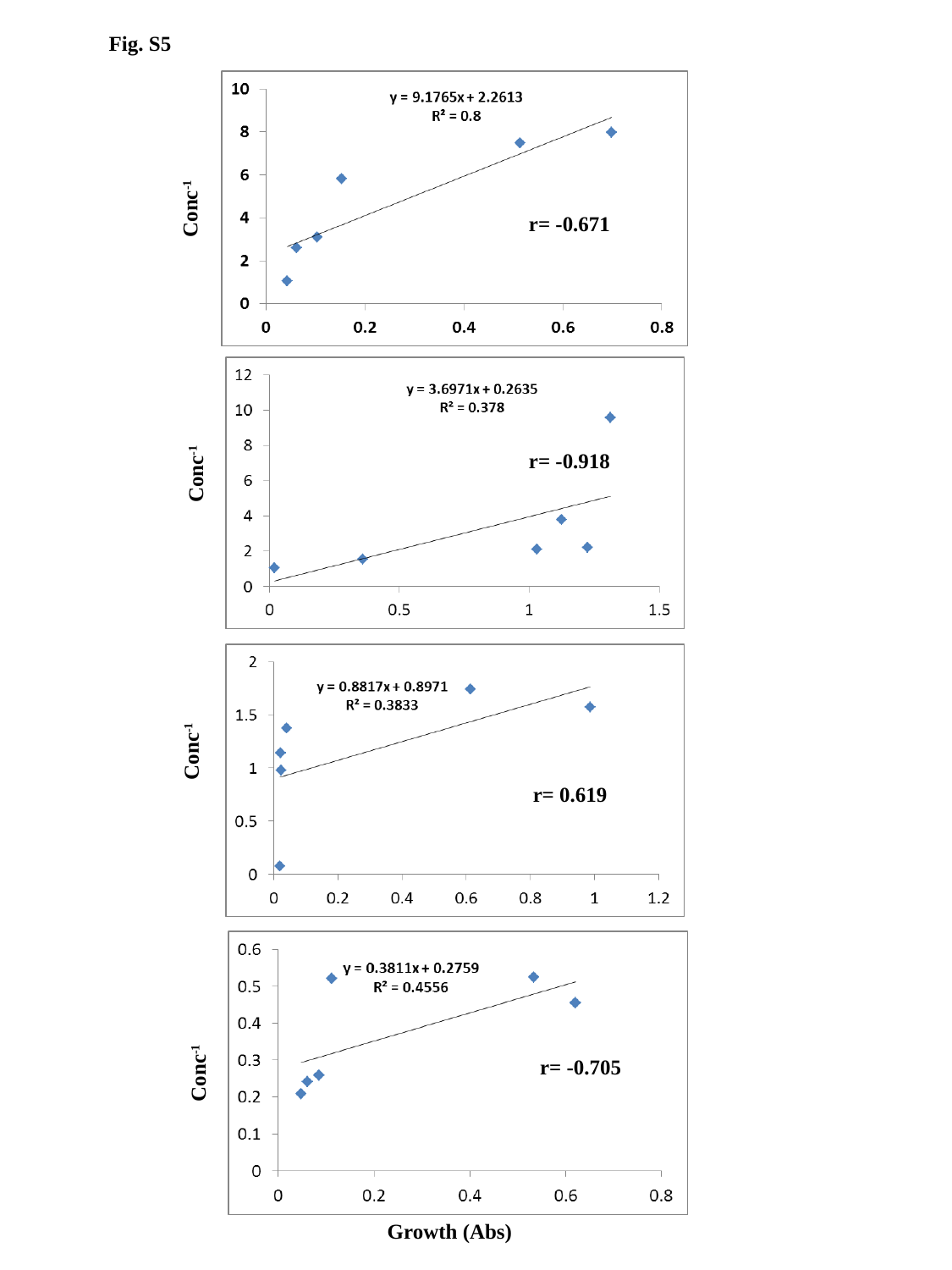

Fig. S5
Conc-1
Conc-1
Conc-1
Conc-1
Growth (Abs)
r= -0.671
r= -0.918
r= 0.619
r= -0.705

## Slide 7
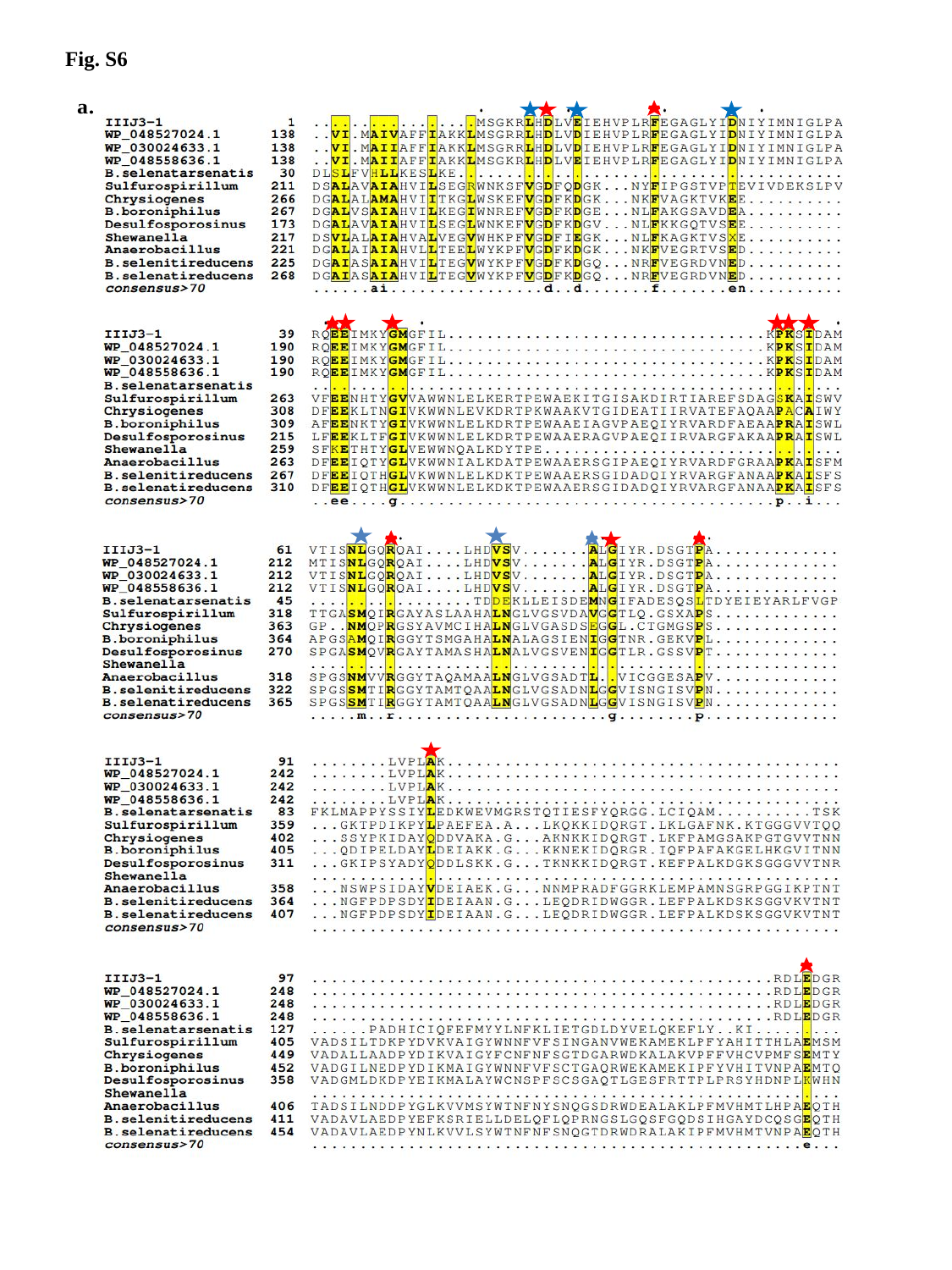

Fig. S6

## Slide 8
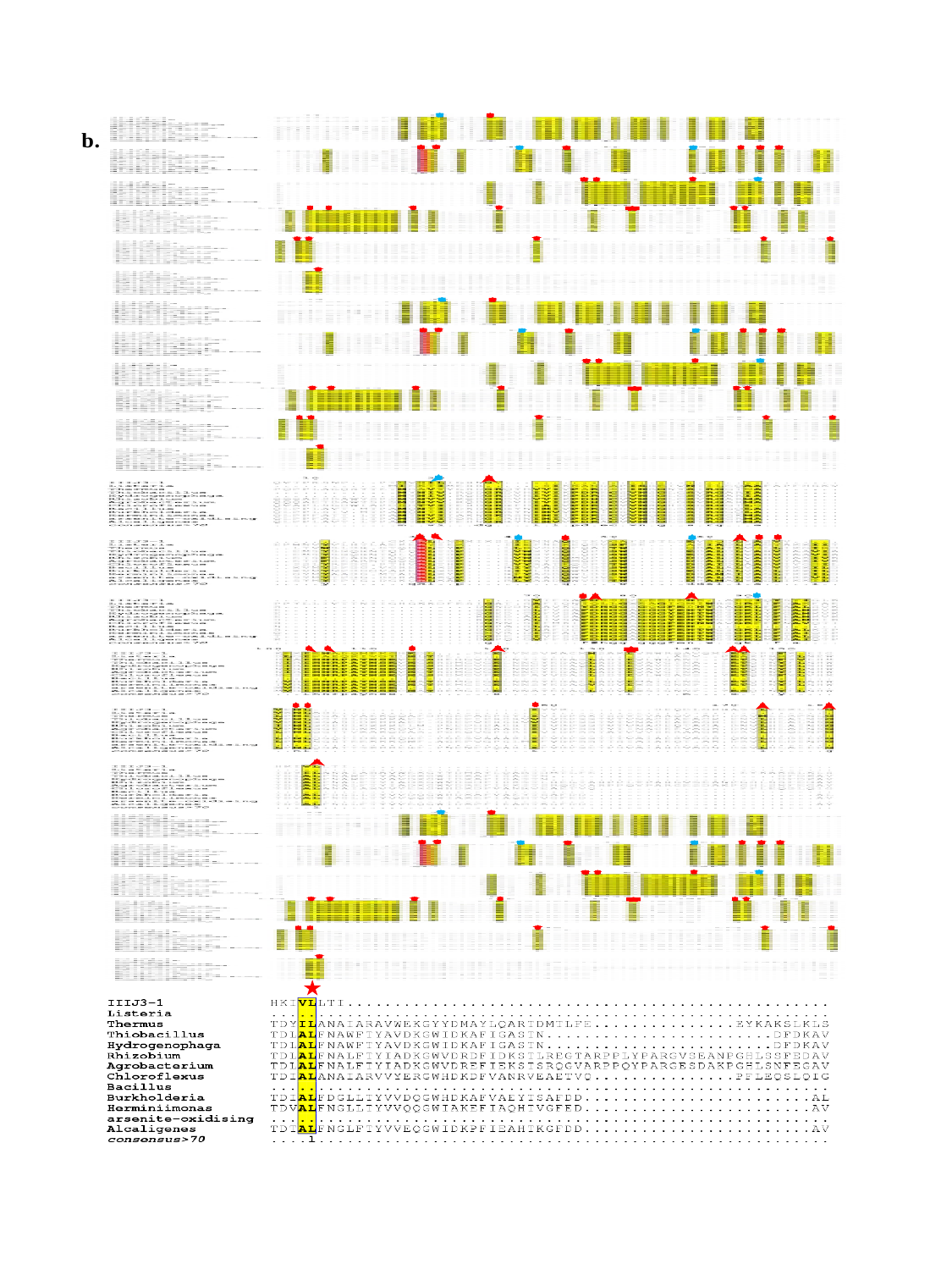

## Slide 9
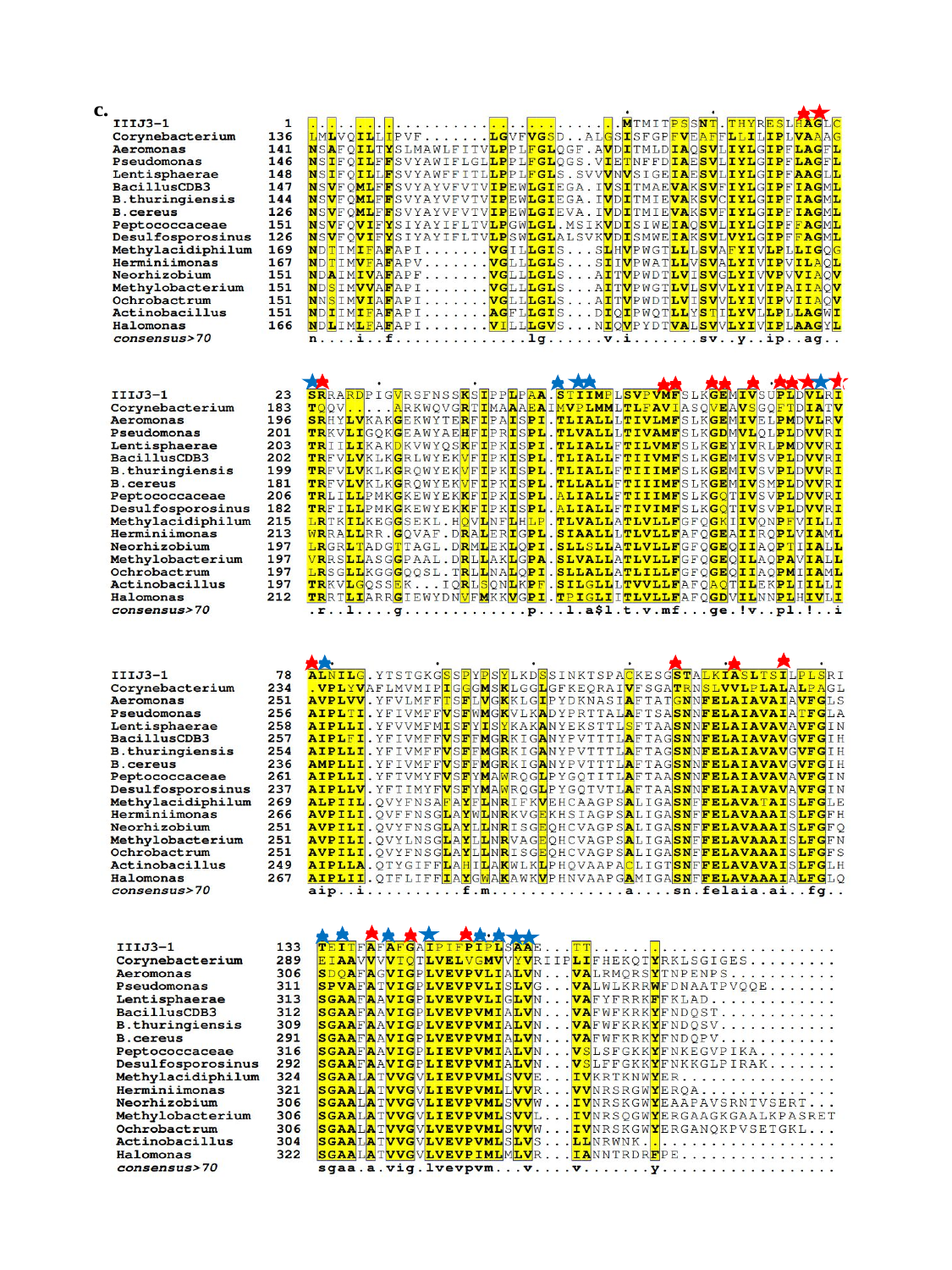

## Slide 10
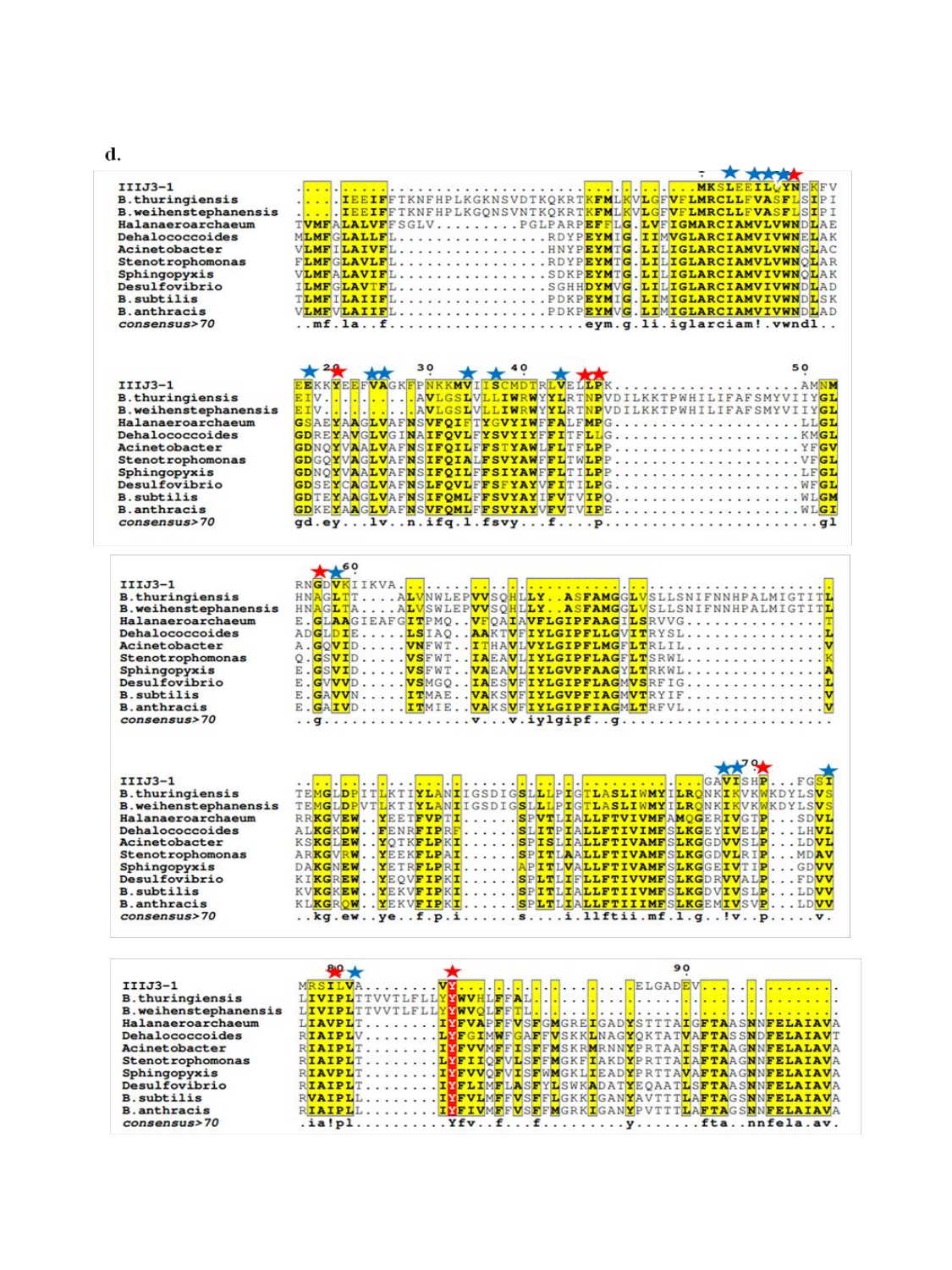

## Slide 11
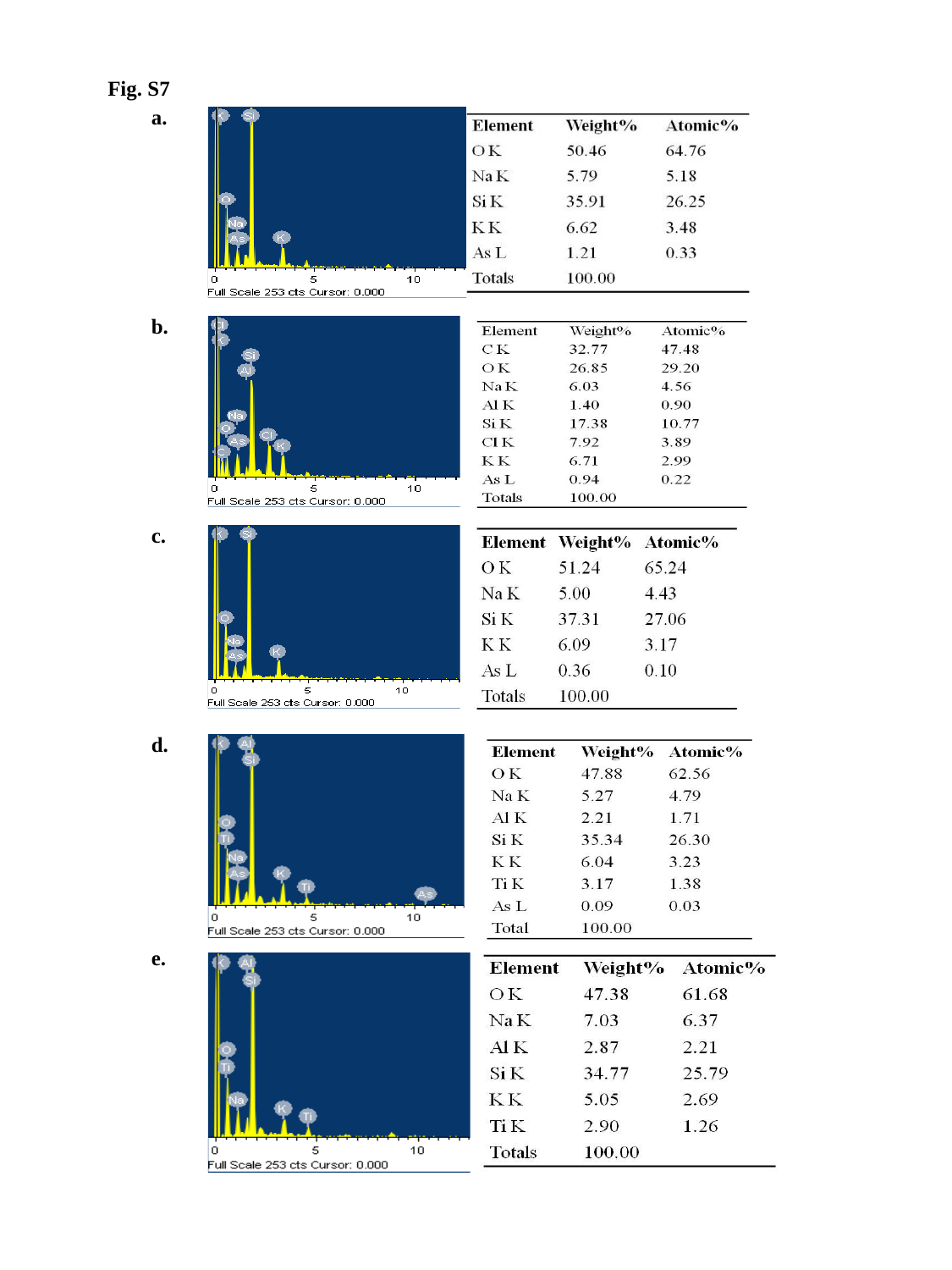

Fig. S7
a.
b.
c.
d.
e.
